# Glutathione modulates the expression of heat shock proteins via the transcription factors BZIP10 and MYB21 in Arabidopsis

**DOI:** 10.1093/jxb/ery166

**Published:** 2018-05-02

**Authors:** Deepak Kumar, Sharmila Chattopadhyay

**Affiliations:** Plant Biology Laboratory, CSIR – Indian Institute of Chemical Biology, Kolkata, India

**Keywords:** *Arabidopsis thaliana*, glutathione, heat shock proteins, proteomics, protoplast, transcription factors

## Abstract

The contribution of glutathione (GSH) in combating environmental stress in plants has long been known. Previous reports have pointed to the involvement of GSH in inducing various heat shock proteins (HSPs), but the molecular mechanism is yet to be explored. Here, we investigate how GSH induces the expression of important HSP genes in Arabidopsis. Expression of HSP genes *BiP3*, *HSP70B*, and *HSP90.1* was positively regulated by GSH, and a promoter activation assay suggested a role for GSH in their induction. Lower expression of *BiP3* and *HSP70B* in the GSH-fed *Atmyb21* mutant and of *HSP90.1* in the GSH-fed *Atbzip10* mutant, in comparison with GSH-fed Col-0, revealed a role for GSH in activating their promoters through the transcription factors MYB21 and BZIP10. Co-transfection of transcription factor mutant protoplasts with transcription factor constructs and HSP promoters confirmed the results. Comparative proteomics also revealed proteins whose expression was controlled by MYB21 and BZIP10 in response to GSH feeding. A co-immunoprecipitation assay demonstrated a role for GSH in modulating the level of interaction of glutathione-*S*-transferase with HSP70. Collectively, our results demonstrate a role for GSH in activating the promoters of *BiP3* and *HSP70B* via MYB21 and of *HSP90.1* via BZIP10.

## Teaser

Glutathione has a role in activating the promoters of heat shock protein genes *BiP3* and *HSP70B* via the transcription factor MYB21 and of *HSP90.1* via the transcription factor BZIP10.

## Introduction

Plants consistently face various stress conditions that adversely affect cellular homeostasis and have harmful effects on their growth, fitness and productivity (Boyer, 1982; Peters *et al.*, 2004; Mickelbart *et al.*, 2015). To protect themselves from extreme environments, plant defense reactions come rapidly into play, within hours or days (Levitt, 1972; Jenks and Hasegawa, 2005). The plant response to different stress conditions is mediated by a complex system of transcription factors (TFs) and regulatory genes that control the stress and defense network (Bray *et al.*, 2000; Cushman and Bohnert, 2000; Suzuki *et al.*, 2005). Although the molecular mechanisms for the expression of stress- and defense-related genes and proteins have been extensively studied globally, with such a complex scenario there is a need for further investigation aimed at the identification of new molecular factors involved.

An important metabolite that plays multiple roles in plant protection and in modulating the stress response is glutathione (GSH), a tripeptide composed of Glu, Cys, and Gly (Marrs and Walbot, 1997; Edwards *et al.*, 2000; Pieterse and Dicke 2007; Noctor *et al.*, 2012; Kovacs *et al.*, 2015). GSH also acts as an important factor determining basal jasmonic acid (JA) gene expression in response to oxidative stress in Arabidopsis (Han *et al.*, 2013). The role of GSH in plant growth and development is well established. Previous reports have indicated its key role in embryo development, meristem development, pollen germination, pollen tube growth, and cell cycle regulation as well (Vernoux *et al.*, 2000; Cairns *et al.*, 2006; Reichheld *et al.*, 2007; Pellny *et al.*, 2009; Zechmann *et al.*, 2011). Earlier reports have shown that plants with an increased GSH level can develop significant tolerance to several environmental stress conditions (Noctor *et al.*, 1998; Gomez *et al.*, 2004; Mullineaux and Rausch, 2005; Ferretti *et al.*, 2009). On the other hand, a reduced GSH level has a negative effect on the biological functions of plant cells, as shown by Arabidopsis plants with low GSH content (<10% of wild type) that were found to be hypersensitive to cadmium stress (Xiang *et al.*, 2001). A GSH mutant line of Arabidopsis, *cad2*, was found to be hypersensitive to metal toxicity (Howden *et al.*, 1995). Another GSH mutant line of Arabidopsis, *pad2.1*, was reported to be deficient in camalexin, which is considered a crucial defense metabolite with a role in limiting the growth of virulent bacteria (Glazebrook and Ausubel, 1994; Parisy *et al.*, 2007). All these GSH mutant lines were reported to have a susceptibility to both biotic and abiotic stress conditions (Ball *et al.*, 2004; Schlaeppi *et al.*, 2008; Kumar *et al.*, 2015). Additionally, as a cofactor GSH plays a key role in the detoxification of methylglyoxal, a cytotoxic compound that is formed as a byproduct of glycolysis under stress conditions (Zang *et al.*, 2001; Singla-Pareek *et al.*, 2003).

In plants a role of GSH has been reported in combating stress through interaction with various stress- and defense-related signaling molecules and its modulation of their pathways (Noctor *et al.*, 2012). It has been demonstrated that GSH mimics salicylic acid (SA) and induces the *PR1* gene by reduction of NPR1 and promoting its recruitment to the nucleus (Mou *et al.*, 2003). A role of GSH in mitigating biotic stress through an NPR1-dependent SA-mediated pathway has been suggested by our group (Ghanta *et al.*, 2011). A recent study by our group has also revealed a role of GSH in regulating 1-aminocyclopropane-1-carboxylate synthase to induce ethylene in response to salinity stress (Datta *et al.*, 2015). GSH also modulates various stress- and defense-related genes by interacting with ABA and ethylene in response to abiotic stress conditions in plants (Yoshida *et al*, 2009; Wei *et al.*, 2015; Kumar *et al.*, 2016).

One of the effects of environmental stress is protein aggregation, which results in protein dysfunction (Lindquist, 1986). Sustaining proteins in their native conformations and preventing the aggregation of non-native proteins are essential for cell survival in response to stress. Heat shock proteins (HSPs) are important chaperones that play a key role in stabilizing proteins and membranes, thereby helping in protein refolding to combat environmental stress (Wang *et al.*, 2004). The correlation between HSP synthesis and an increase in thermotolerance in plants is well known. Furthermore, previous reports have shown that expression of HSPs is associated with the intensity of the stress that they are naturally exposed to (Howarth and Ougham, 1993; Feder and Hofmann, 1999). The interaction of HSPs such as HSP60, HSP70, and HSP90 with a wide range of co-chaperone proteins that regulate their activity has been reported (Bukau and Horwich, 1998; Buchner, 1999; Frydman, 2001). *HSP70*-overexpression lines of *Nicotiana* and Arabidopsis showed tolerance to salt, water, and high-temperature stress (Leborgne-Castel *et al.*, 1999; Sugino *et al.*, 1999; Alvim *et al.*, 2001; Ono *et al.*, 2001; Sung and Guy, 2003). Plant HSPs acts in response to a broad range of environmental stresses, including heat, cold, drought, salinity, and oxidative stress (Wang *et al.*, 2004). HSP90 activity is also responsible for brassinosteroid signaling via trafficking of BIN2–HSP90 complexes into the cytoplasm (Samakovli *et al.*, 2014).

In our previous work we have reported an elevated level of HSP expression in a GSH-fed line of Arabidopsis (Sinha *et al.*, 2015). In the GSH-overexpression line of *Nicotiana*, HSP70 was notably up-regulated as well (Ghanta *et al.*, 2011). We have also found reduced levels of expression of the same members of the HSP family in combined stress-treated *pad2.1*, a GSH mutant of Arabidopsis, which were previously found to be up-regulated in GSH-fed Arabidopsis (Kumar *et al.*, 2015). Together, previous reports have pointed towards the involvement of GSH in inducing HSPs, which plays a vital role in combating various stresses in the plant system. Here, we demonstrate the molecular mechanism through which GSH induces the expression of these HSPs. Our results unravel the function of GSH in modulating the efficiency of interaction of HSP70 with other proteins to combat environmental stresses *in planta*.

## Materials and methods

### Plant materials

All Arabidopsis seeds were obtained from the Nottingham Arabidopsis Stock Centre (NASC). SALK_106031:*Atbzip10*, N6241:*AtAP2*, SALK_042711C:*Atmyb21*, N8052:*Atein3*, and N3804:*pad2.1* are mutants of BZIP10, APETALA2, MYB21, EIN3, and GSH respectively. The *pad2.1* mutant line of Arabidopsis has a mutation of the first enzyme, viz. γ-glutamylcysteine synthetase (γ-ECS), of the glutathione pathway, thus reducing the GSH content by 80% compared with the wild type (Parisy *et al.*, 2007; Zechmann *et al.*, 2008). Arabidopsis Col-0 ecotype, the wild type, and *AtECS1* (a *γ-ECS* overexpression line) were used also. Surface-sterilized seeds were grown in Murashige and Skoog (MS) medium, and maintained in a growth chamber (Eyela) at 22 °C under a 16 h light–8h dark cycle. The collective leaf structures of 3- and 4-week-old seedlings were harvested for RNA and protein isolation.

### Chemical treatment of seedlings

Three-week-old seedlings were used for all feeding experiments. Buthionine sulphoximine (BSO) is an important inhibitor of the first enzyme in GSH biosynthesis thus leading to a strong decrease in GSH level in Arabidopsis (Meyer and Fricker, 2002). For GSH and BSO feeding, seedlings were fed separately with 100 μM GSH and 1 mM BSO solutions for 24, 48, 72, and 96 h, as described previously (Sinha *et al.*, 2015; Datta *et al.*, 2015). Both dithiothreitol (DTT) and β-mercaptoethanol (β-ME) are strong reducing agents that produce a reducing environment in plant cells (Lindermayr *et al.*, 2010). For DTT and β-ME feeding, seedlings were fed with 5 mM freshly prepared DTT and β-ME solutions.

### RNA extraction and quantitative RT-PCR analysis

Total RNA from the collective leaf structures of Arabidopsis was isolated using the TriZol method. Quantitative RT-PCR was performed using the total RNA of both control and mutant lines. One microgram of total RNA of each sample was reverse-transcribed using the RevertAid first strand cDNA synthesis kit (Fermentas) using oligo(dT) as a primer. Using the Roche LightCycler 96 System (Roche) with FastStart Essential DNA Green Master (Roche), quantitative RT-PCR was performed. The selected genes for which quantitative RT-PCR was performed are listed in Supplementary Table S1 (at *JXB* online). PCR amplification was performed for 40 cycles at 94 °C, 30 s and 60 °C, 2.5 min with a preceding initial denaturation of 30 s at 95 °C. Both *actin* and *tubulin* were used as the reference gene.

### Protein isolation and western blotting

Total protein was isolated from about 1 mg of leaf tissue of all plant samples by using the phenol extraction method. The finely ground tissue was suspended in extraction buffer (700 mM sucrose, 500 mM Tris–HCl, pH 7.5, 50 mM EDTA, 100 mM KCl, 2% (w/v) β-ME, 1 mM phenylmethylsulfonyl fluoride) and protein extraction was completed following a standard protocol. The extracted protein was further dissolved in resuspension buffer consisting of 7 M urea, 2 M thiourea, 4% 3-[(3-cholamido propyl)-dimethylammonio]-1-propane sulfonate (CHAPS), 20 mM DTT and 1% (w/v) Bio-Lyte (3/10) ampholyte (Bio-Rad) as described previously (Kumar *et al.*, 2015). Protein quantity was measured by using the Bradford method.

From the isolated total proteins from Col-0, *AtECS1*, and mutants, the protein bands of HSP70 and HSP90.1 were identified using rabbit polyclonal anti-HSP70 and HSP90.1 antibody as the primary antibodies against Arabidopsis proteins, and horseradish peroxidase-conjugated anti-rabbit IgG was used as secondary antibody (Agrisera). Immunoreactive proteins were visualized by using the Pico chemiluminescent substrate (Pierce).

### Promoter analysis using protoplast isolation and transfection assay

The promoter regions of the genes *BiP3*, *HSP70B*, and *HSP90.1*, i.e. the intergenic region upstream to 938, 420, and 693 bases, respectively, of the transcription start site were cloned into *pCAMBIA1303* plasmid with the green fluorescent protein (GFP) gene under control of these promoters. As a result, *proBiP3:GFP*, *proHSP70B:GFP*, and *proHSP90.1:GFP* constructs were developed. Protoplasts were isolated from the leaves of 3-week-old Col-0, *AtECS1*, and *pad2.1* seedlings. Isolated protoplasts were transfected with *proBiP3:GFP*, *proHSP70B:GFP*, and *proHSP90.1:GFP* constructs separately by using the polyethylene glycol (PEG)–CaCl_2_ method (Yoo *et al.*, 2007). After transfection the protoplasts were maintained for 6 h at room temperature. Visualization of GFP expression levels in isolated protoplasts for each construct was performed using a confocal laser scanning microscope. We quantified the GFP fluorescence of at least three protoplasts at ×60 magnification for each experiment. We also checked the fluorescence in almost all the protoplasts (*n*≥10) present at ×20 magnification through confocal microscopy. GFP fluorescence in transfected protoplasts was measured using ImageJ/Fiji software. Through the software we checked the fluorescence of the whole area of an individual protoplast at ×20 and ×60 magnification. After that we divided the fluorescence of all the protoplast by the area of the protoplast, giving the fluorescence per unit area of an individual protoplast. This method was applied to all the protoplasts present at ×20 and ×60 magnification. After that we took the mean of the fluorescence/area of all the protoplasts present in selected fields; the value of the fluorescence/area of the *HSP*-promoter transfected protoplasts of altered GSH plant samples was normalized with the value of the fluorescence/area of control protoplast (GFP expression in transfected protoplast of Col-0 with *pCAMBIA1303* construct devoid of *HSP* promoter).

### *In silico* identification of promoter-sequence-binding TFs

Detection of various TFs that could bind to the promoter sequence of *BiP3*, *HSP70B*, and *HSP90.1* was performed by using Plant Promoter Analysis Navigator (PlantPan; http://PlantPan2.itps.edu.tw;Chow *et al.*, 2016).

### Procurement of TF mutants, GSH feeding and checking the expression of HSPs

On the basis of *in silico* identification of HSP promoter sequence-binding TFs, we procured TF mutants from NASC and germinated them as mentioned above. Again, 3-week-old seedlings were fed with 100 μM GSH. HSP expression was determined in all the GSH-fed TF mutants at both gene and protein levels by using the method described above.

### Protoplast transfection of TF mutant lines

Protoplasts of *Atmyb21* and *Atbzip10* mutants were transfected with *proBiP3:GFP*, *proHSP70B:GFP*, and *proHSP90.1:GFP* constructs. GFP expression was visualized in the GSH-fed transfected protoplast by confocal microscopy.

### Protoplast co-transfection assay

From Arabidopsis cDNA, the coding DNA sequence (CDS) of *myb21* was cloned into the pBI121 vector. As a result, the *proCaMV35S:MYB21* construct was developed. The The *Discosoma*-specific red fluorescent protein (*DsRed*) sequence of the pDsRed-Monomer-N1 vector (Clontech) was cloned into the proCaMV35S:MYB21 vector, which resulted in the development of *proCaMV35S:MYB21-DsRed* constructs. The expression of both *myb21* and *DsRed* was under the control of the 35SCaMV constitutive promoter. Isolation of protoplasts was performed from the leaves of 3-week-old *Atmyb21*. By using the PEG–CaCl_2_ method, the *proCaMV35S:MYB21-DsRed* construct was co-transfected with *proBiP3:GFP* or *proHSP70B:GFP* in Col-0 and *Atmyb21* mutant protoplasts. GSH of concentration 100 μM was added to the protoplast-maintenance medium after the transaction and maintained at room temperature for about 6 h. In the same way *bzip10* CDS isolated from Arabidopsis was cloned in the pAM-PAT-YFP vector under the control of 35SCaMV constitutive promoter. The resulting construct was *proCaMV35S:YFP-BZIP10*. In the next step the protoplasts of Col-0 and *Atbzip10* lines were co-transfected with *proCaMV35S:YFP-BZIP10* and *proHSP90.1:GFP*. Again GSH of 100 μM was added to the protoplast-maintenance medium for about 6 h. By using a confocal laser scanning microscope and a fluorescence microscope, GFP, DsRed and yellow fluorescent protein (YFP) expression levels were examined.

### Homology modeling for molecular docking

A 3D model of MYB21 was generated by using the homology modeling program Swiss-Model (Biasini *et al.*, 2014; Guex *et al.*, 2009). At the start, the potentially related amino acid sequences of MYB21 were searched. To evaluate the model folding, the resulting model was subjected to PROCHECK (Laskowski *et al.*, 1993). The active-site groove volume of the enzyme was measured through CASTp calculation (Dundas *et al.*, 2006).

### Two-dimensional gel electrophoresis

One gram of total protein was isolated from Col-0, GSH-fed Col-0, GSH-fed *myb21* and GSH-fed *bzip10* mutant lines of Arabidopsis. The extracted protein was then resuspended in IEF buffer consisting of 7 M urea, 2 M thiourea, 4% CHAPS, 20 mM DTT and 1% (w/v) Bio-Lyte (3/10) ampholyte (Bio-Rad) as before. Quantification of protein was performed by using the Bradford method. Protein of approximately 600 μg was re-hydrated in an immobilized pH gradient strip (7 cm; pH 4–7; GE) for 12 h. Isoelectric focusing was performed as follows: 250 V for 30 min, 8000 V for 2 h, 8000 V for 26 000 V h, 750 V for 1 h on a Bio-Rad PROTEAN IEF Cell system. After 15 min equilibration of focused strips in calibration buffers I and II (Bio-Rad) second-dimension gels were run by using 12% SDS polyacrylamide gels and staining with colloidal Coomasie Brilliant Blue (CBB) G-250.

### Image analysis

The gel images were analysed with a Versa doc image system (Bio-Rad) and PD Quest software version 8.0.1 (Bio-Rad). Spot detection was performed by matching the gels automatically with manual verification. The spots detected in at least two replicate gels were selected for annotation.

### Tryptic in-gel digestion and mass spectrometry analysis

Manually excised differentially accumulated protein spots were digested with trypsin (in-gel trypsin digestion kit, Pierce) using the manufacturer’s protocol. The mass spectrometric analysis of the samples was performed by using Zip-Tip μ-C18 (Millipore) and a 4800 matrix-assisted laser desorption/ionization time-of-flight/time-of-flight (MALDI TOF/TOF) analyser (Applied Biosystems). By using STRING 10 software, the protein interactions between identified differentially accumulated proteins in response to GSH treatment were recognized. The expression of some differentially accumulated proteins was validated by immunoblotting.

### Protein immunoprecipitation and western blot analysis

Total protein was isolated from Col-0, *pad2.1*, and *AtECS1*. About 3 mg of total proteins was inoculated in protein isolation buffer containing protein A (magnetic beads, Millipore) and rabbit polyclonal anti-HSP70 primary antibody (Agrisera) overnight at 4 °C. After that the protein A solution was centrifuged and the pellet was taken. Extracted pellet was washed three times by dissolving in protein isolation buffer. Twenty microliters of protein loading dye (2% SDS, 100 mM Tris–HCl pH 7.5, 10% glycerol, 0.5 mM EDTA, 10 mM DTT) was added to the washed pellet and heated at 70 °C for 10 min. From the upper eluted liquid samples, western blotting was performed by using rabbit polyclonal anti-glutathione-*S*-transferase (GST) phi primary antibody (Agrisera). As a control the expression of HSP70 was also checked by using the same eluted sample.

### Statistical analysis

All the experiments were performed in triplicate and the data presented as the mean±standard error (SE) to compare the relative gene and protein expression profiles of Col-0 and mutant lines. For statistical analysis, ANOVA followed by the Student–Newman–Keuls multiple comparison test was used (GraphPad InStat software, v. 3.1). *P*<0.05, 0.01 or 0.001 was considered to be statistically significant.

## Results

### Altered GSH conditions regulate the expression of HSP genes

**Fig. 1.**
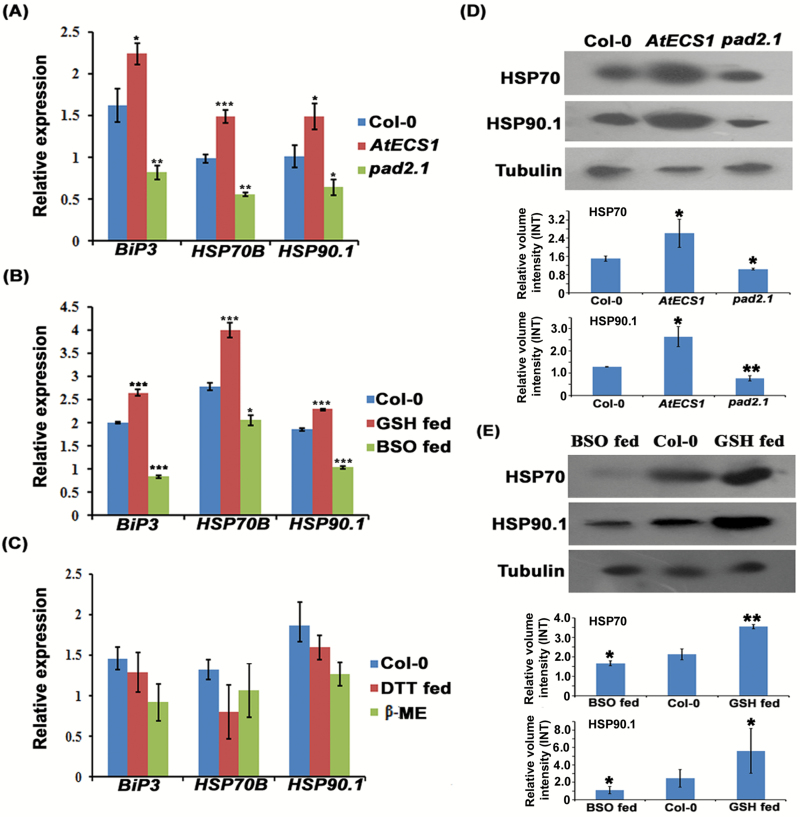
Effect of altered GSH level on the expression of HSPs at transcript and protein levels in three Arabidopsis lines, viz. transgenic *AtECS1*, GSH-mutant *pad2.1*, and wild type Col-0. (A) In *AtECS1* transgenic line the expression of *BiP3*, *HSP70B*, and *HSP90.1* were noticeably up-regulated and the same genes were found to be down-regulated in the *pad2.1.* (B) Three-week-old Col-0 plants were treated with 100 µM GSH and 1 mM BSO for 72 h. All three HSP genes were significantly up-regulated in GSH-fed Col-0 but the same genes were observed to be down-regulated in BSO fed Col-0. See Supplementary Fig. S1 for quantitative RT-PCR expression analysis. (C) For developing a reduced environment in the plant cells, Col-0 was treated with β-ME and DTT. No significant change in the expression of HSP genes was noticed in β-ME- and DTT-treated Col-0. Data are presented as mean ±SE (*n*=3). *P<0.05, **P<0.01, ***P<0.001. (D) Western blotting was performed from 3-week-old plant samples. Both HSP70 and HSP90.1 were up-regulated in *AtECS1* and down-regulated in the *pad2.1* line. (E) Elevated expression level of HSP70 and HSP90.1 proteins was noticed in GSH-fed Col-0 and reduced expression level of the same proteins were observed in BSO-fed Col-0. All the experiments were performed in triplicate. Tubulin: loading control for normalization. INT, unit of volume intensity. (This figure is available in color at *JXB* online.)

The expression of the HSP genes (*HSP*s) *BiP3*, *HSP70B*, and *HSP90.1* was determined in the *γ-ECS*-overexpressing transgenic line *AtECS1*, GSH mutant line *pad2.1*, and wild type Col-0. Expression of *BiP3*, *HSP70B*, and *HSP90.1* was found to be greater in the *AtECS1* line, whereas a lower expression level was noted in *pad2.1* in comparison with Col-0 at both gene and protein levels (Fig. 1A, D; Supplementary Figs S1, S2). A similar result with greater expression of *HSP*s in the GSH-fed line of wild type Col-0 and lower expression in BSO-fed Col-0 in comparison with untreated Col-0 was observed at both gene and protein levels (Fig. 1B, E; Supplementary Figs S1–S3). To check the effect of a reducing environment on the expression of these *HSP*s, wild type Col-0 was treated with β-ME and DTT; no significant change in the expression of these *HSP*s was noticed in the treated wild type Col-0 in comparison with untreated Col-0 (Fig. 1C; Supplementary Fig. S1C). These results point towards a role of GSH in inducing these *HSP*s.

### GSH has a role in activating the promoters of *HSP*s

**Fig. 2.**
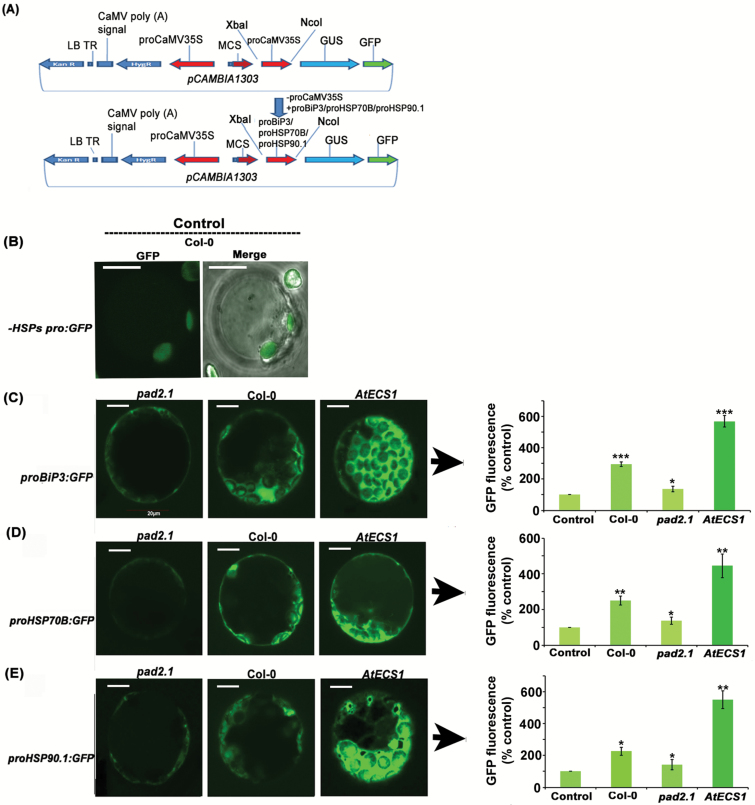
Promoter activation analysis of *proBiP3*, *proHSP70B*, and *proHSP90.1* in response to altered GSH conditions. Leaf protoplasts of Col-0, *AtECS1*, and *pad2.1* were transfected with *proBiP3:GFP*, *proHSP70B:GFP*, and *proHSP90.1:GFP.* Promoter activity was monitored by checking the fluorescence of GFP in the transfected protoplast under a confocal microscope. (A) Linear map of pCAMBIA1303 vector in which the promoter sequences of *BiP3*, *HSP70B*, and *HSP90.1* were inserted upstream of *GFP*. LB-TR, left border T-DNA repeat. (B) Control: GFP expression in Col-0 protoplasts transfected with pCAMBIA1303 plasmid, which was devoid of the HSP promoter (−*HSPspro:GFP*). (C) Elevated GFP fluorescence was observed in *AtECS1* protoplasts transfected with *proBip3:GFP*. Reduced GFP expression was noticed in *pad2.1*-transfected protoplasts. (D) Higher GFP fluorescence was observed in *AtECS1* protoplasts transfected with *proHSP70B:GFP* and less GFP fluorescence was found in transfected protoplasts of *pad2.1*. (E) Similarly, more GFP fluorescence was noted in *AtECS1* protoplasts transfected with *proHSP90.1:GFP* and less fluorescence was observed in transfected protoplasts of *pad2.1.* GFP fluorescence was noted at 488 nm. Scale bar: 10 µm. GFP fluorescence in control protoplast has been normalized (100%). Data are presented as mean ±SE (*n*=3 at ×60 magnification, *n*≥10 at ×20 magnification). ANOVA followed by Student–Newman–Keuls multiple comparison test was used (GraphPad InStat software, v. 3.1). *P<0.05, **P<0.01, ***P<0.001.

GFP expression was noticed in the cytoplasm of the protoplasts with the promoter activation of these *HSP*s. As a control for GFP expression, Col-0 protoplasts were transfected with the pCAMBIA1303 plasmid, which lacked an HSP promoter (−*HSPspro:GFP*), and GFP expression was estimated by confocal microscopy (Fig. 2B). We observed the highest level of GFP expression in the *AtECS1* protoplast transfected with *proBiP3:GFP*, *proHSP70B:GFP*, and *proHSP90.1:GFP* and observed the least expression of GFP in the *pad2.1* protoplast transfected with these constructs (Fig. 2C–E; Supplementary Figs S4, S5; Supplementary Table S2). These findings further support a role of GSH in inducing these *HSP*s by activating their promoters.

### Identification of HSP promoter sequence-binding TFs

By using PlantPan software (Chow *et al.*, 2016) we found that TFs such as EIN3, APETALA2, BZIP10, and MYB21 had a large number of binding sites in the promoter sequence of *BiP3*, *HSP70B*, and *HSP90.1* (see Supplementary Fig. S6). For BZIP10, MYB21, APETALA2, and EIN3, the conserved binding sequences of the promoters of *HSP*s were ACGT, ACCTT/GAAC, ACCG/CGAC, and TACA/GTAT, respectively. On this basis, Arabidopsis mutant lines of these TF genes were used.

### Determining the expression and promoter activity of *HSP*s in TF mutant lines in response to GSH feeding

**Fig. 3.**
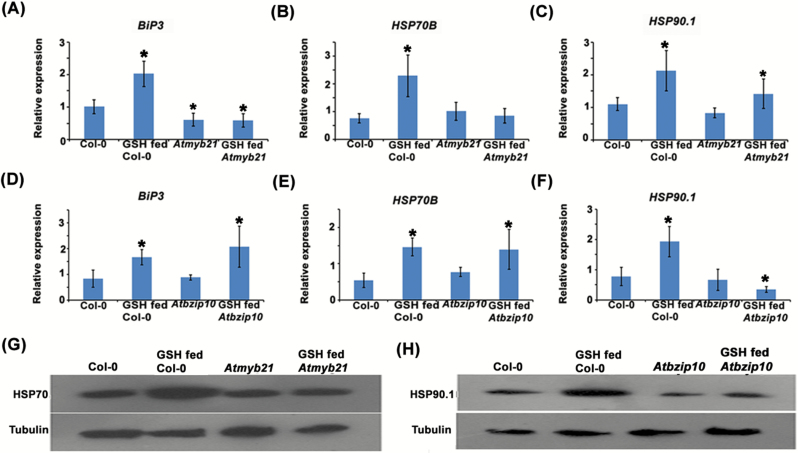
Effect on the expression of HSPs in GSH-fed *Atmyb21* and *Atbzip10* mutant lines. (A–C) Expression of *HSP*s in Col-0, GSH-fed Col-0, *Atmyb21*, and GSH-fed *Atmyb21* mutant lines at transcript level. (A, B) There was no significant increase in the expression of *BiP3* and *HSP70B* in GSH-fed *Atmyb21* mutant compared with GSH-fed Col-0, suggesting a role of MYB21 in inducing *BiP3* and *HSP70B* in response to GSH feeding. (D–F) The expression of *HSP*s in Col-0, GSH-fed Col-0, *Atbzip10*, and GSH-fed *Atbzip10* mutant lines at transcript level. (F) Reduced expression of *HSP90.1* in GSH-fed *Atbzip10* when compared with GSH-fed Col-0, pointing towards a role of BZIP10 in inducing *HSP90.1* in response to GSH. See Supplementary Fig. S6 for determination of the binding sequences of TFs at the promoter region of HSPs. Supplementary Fig. S8 shows the transcript results for the HSPs in other TF mutant lines. Data are presented as mean ±SE (*n*=3). ANOVA followed by Student–Newman–Keuls multiple comparison test was used (GraphPad InStat software, v. 3.1). *P<0.05, **P<0.01, ***P<0.001. (G, H) Western blot analysis also revealed lesser expression of HSP70 and HSP90.1 in GSH-fed *Atmyb21* and GSH-fed *Atbzip10* mutants, respectively, in comparison with GSH-fed Col-0, which further validated the transcript result. Tubulin: loading control for normalization. (This figure is available in color at *JXB* online.)

TF mutant lines *Atein3*, *Atapetala2*, *Atbzip10*, and *Atmyb21* were treated with 100 μM GSH for 72 h, and the expression of *HSP*s in these treated mutant lines was compared with GSH-fed Col-0. The results revealed lower expression of *BiP3* and *HSP70B* in the GSH-fed *Atmyb21* mutant in comparison with GSH-fed Col-0 at both gene and protein levels (Fig. 3A, B, G; Supplementary Fig. S7A). On the other hand, lower expression of *HSP90.1* was noticed in the GSH-fed *Atbzip10* mutant line in comparison with GSH-fed Col-0 at both gene and protein levels (Fig. 3F, H; Supplementary Fig. S7A). We found insignificant change in the expression of these *HSP*s in GSH-fed *Atein3* and *AtAP2* in comparison with GSH-fed Col-0 (Supplementary Fig. S8).

**Fig. 4.**
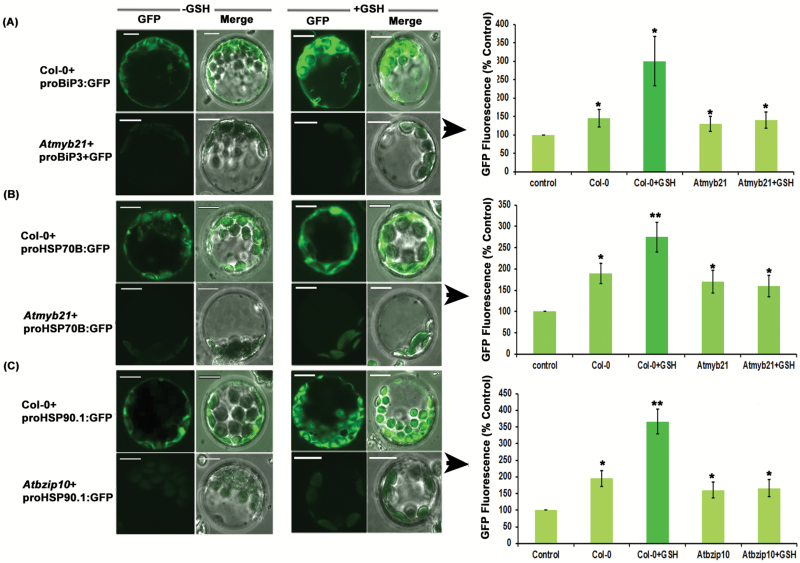
Effect on the promoter activity of HSPs in GSH-fed *Atmyb21* and *Atbzip10* mutant lines. (A, B) Protoplasts isolated from *Atmyb21* transfected with *proBiP3:GFP* and *proHSP70B:GFP.* (C) Protoplasts of *Atbzip10* transfected with *proHSP90.1:GFP. GFP* expression was monitored by confocal microscopy with or without GSH. Reduced GFP fluorescence was observed in GSH-fed transfected protoplasts of *Atmyb21* and *bzip10* in comparison with transfected GSH-fed Col-0. Scale bar: 10 µm. GFP fluorescence in control protoplast (mentioned in Fig. 2B) has been normalized (100%). Data are presented as mean ±SE (*n*≥3). ANOVA followed by Student–Newman–Keuls multiple comparison test was used (GraphPad InStat software, v. 3.1). *P<0.05, **P<0.01, ***P<0.001.

The protoplasts of Col-0 and mutant lines were transfected with *proBiP3:GFP*, *proHSP70B:GFP*, and *proHSP90.1:GFP*, and the expression of GFP in the cytoplasm was observed in the GSH-fed transfected protoplasts by confocal microscopy. The results revealed lower expression of GFP in the GSH-fed *Atmyb21* mutant line protoplast transfected with *proBiP3:GFP* and *proHSP70B:GFP* in comparison with GSH-fed Col-0 protoplast transfected with these constructs (Fig. 4A, B). Similarly, we also observed lower GFP expression in the GSH-fed *Atbzip10* mutant line protoplasts transfected with *proHSP90.1:GFP* in comparison with GSH-fed transfected protoplast of Col-0 (Fig. 4C). These results pointed towards a role for MYB21 in activating the promoters of *BiP3* and *HSP70B* in response to GSH. On the other hand, these results also suggested a role for BZIP10 in activating the promoter of *HSP90.1* in response to the elevated level of GSH in the plant cells.

### Co-transfection assay confirmed the function of MYB21 and BZIP10 in the activation of promoters of *HSP*s

**Fig. 5.**
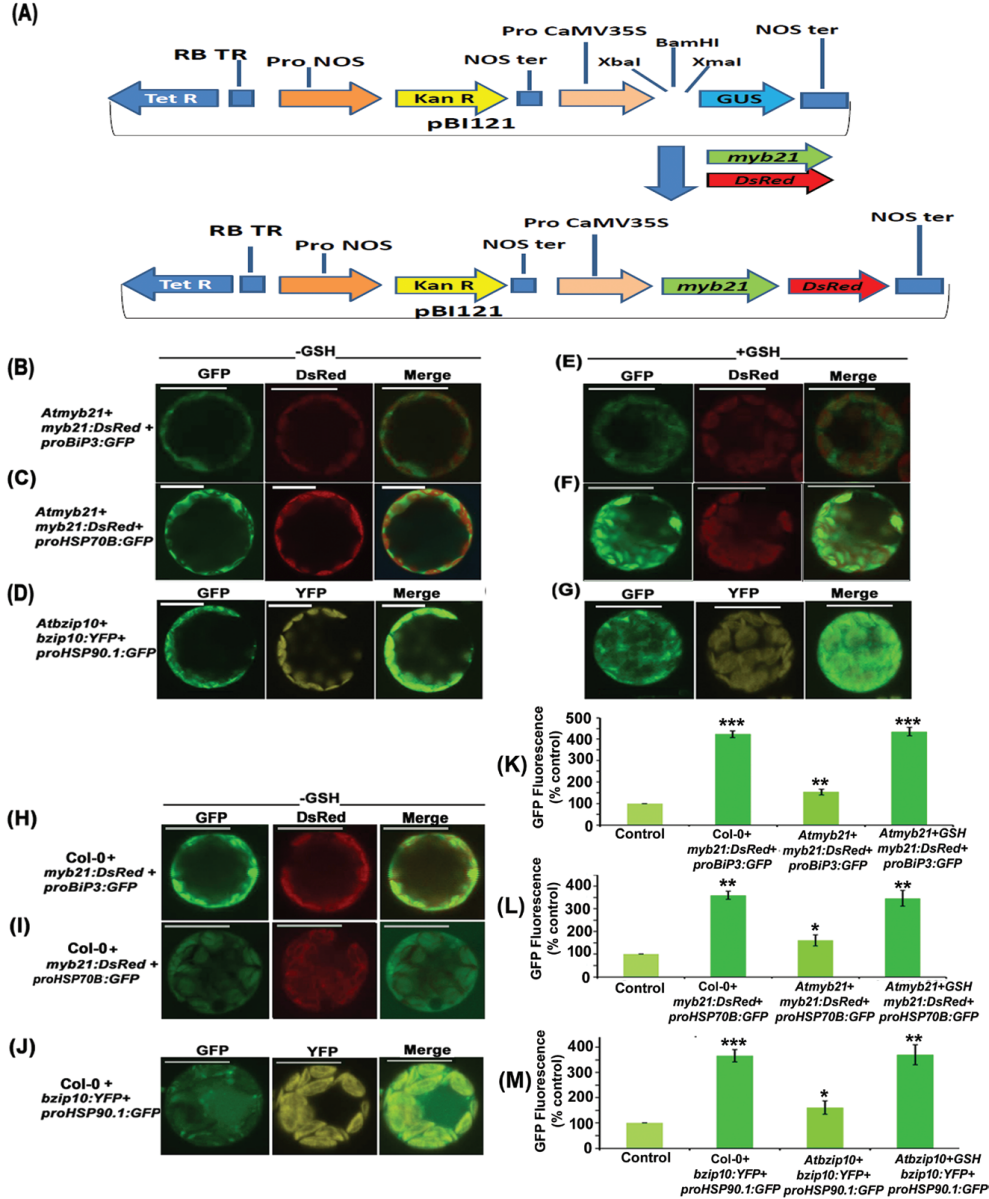
Promoter activity analysis of *proBiP3*, *proHSP70B*, and *proHSP90.1* in *Atmyb21* and *Atbzip10* protoplasts. (A) Linear map of pBI121 vector in which sequences of *MYB21* and *DsRed* were inserted under the control of CaMV35S promoter. RB-TR, right border T-DNA repeat; NOS, nopaline synthase. (B) Protoplasts of *Atmyb21* were co-transfected with *proCaMV35S:myb21-DsRed* and *proBiP3:GFP.* (C) *Atmyb21* protoplasts were co-transfected with *proCaMV35S:MYB21-DsRed* and *proHSP70B:GFP.* See Supplementary Fig. S9 for fluorescence microscopy result. (D) Protoplasts of *Atbzip10* co-transfected with *proCaMV35S:BZIP10-YFP* and *proHSP90.1:GFP.* GFP, DsRed and YFP expression were monitored under a confocal microscope in GSH-fed and -unfed conditions. DsRed fluorescence reported the expression of MYB21. (E, H, K) After co-transfecting *Atmyb21* protoplasts with *proCaMV35S:MYB21-DsRed* and *proBiP3:GFP*, significant increase in GFP fluorescence was observed in response to GSH treatment, indicating the restoration of GFP expression, like in cotransfected protoplast of Col-0. (F, I, L) Co-transfection of *Atmyb21* protoplasts with *pCaMV35S:MYB21-DsRed* and *proHSP70B:GFP* significantly elevated *GFP* expression in response to GSH treatment. (G, J, M) Similarly, after co-transfecting *Atbzip10* protoplasts with *pCaMV35S:BZIP10-YFP* and *proHSP90.1:GFP*, significant increase in GFP fluorescence was observed in response to GSH feeding. Scale bar: 20 µm. GFP fluorescence in control protoplasts (mentioned in Fig. 2B) has been normalized (100%). Data are presented as mean ±SE (*n*=3). ANOVA followed by Student–Newman–Keuls multiple comparison test was used (GraphPad InStat software, v. 3.1). *P<0.05, **P<0.01, ***P<0.001.

The findings mentioned above encouraged us to perform a co-transfection assay in *Atmyb21* and *Atbzip10* mutant protoplasts. As a complementation test for this result, *myb21* and *bzip10* genes were cloned in proCaMV35S:DsRed and pAM-PAT-YFP vector, respectively, under the control of the CaMV35S promoter. The resulting constructs were *proCaMV35S:MYB21-DsRed* and *proCaMV35S:BZIP10-YFP*. After transfecting the *proCaMV35S:MYB21-DsRed* and *proCaMV35S:BZIP10-YFP* constructs in the protoplasts of *Atmyb21* and *Atbzip10* mutants, respectively, we observed the restoration of the *myb21* mutant by determining the expression of DsRed fluorescence. After co-transfecting the construct *proCaMV35S:MYB21-DsRed* with *proBiP3:GFP* or *proHSP70B:GFP* in GSH-fed *Atmyb21* protoplasts, we observed restored GFP expression, like the co-transfected protoplasts of Col-0 (Fig. 5B, C, E, F, H, I, K, L; Supplementary Fig. S9). This result suggested a role for MYB21 in activating the promoters of *BiP3* and *HSP70B* in the GSH-fed condition. Similarly, when the protoplasts of *Atbzip10* were co-transfected with *proCaMV35S:BZIP10-YFP* and *proHSP90.1:GFP* in the GSH-fed condition, the expression of GFP was restored in the *bzip10* protoplast (Fig. 5D, G, J, M). This result further indicated that *bzip10* plays a key role in activating the *HSP90.1* promoter. Together, our results pointed towards a role for MYB21 in activating the promoters of *BiP3* and *HSP70B* and for BZIP10 in activating the promoter of *HSP90.1* in response to an elevated level of GSH.

### GSH induces the expression of *myb21* without executing its post-translational modification

**Fig. 6.**
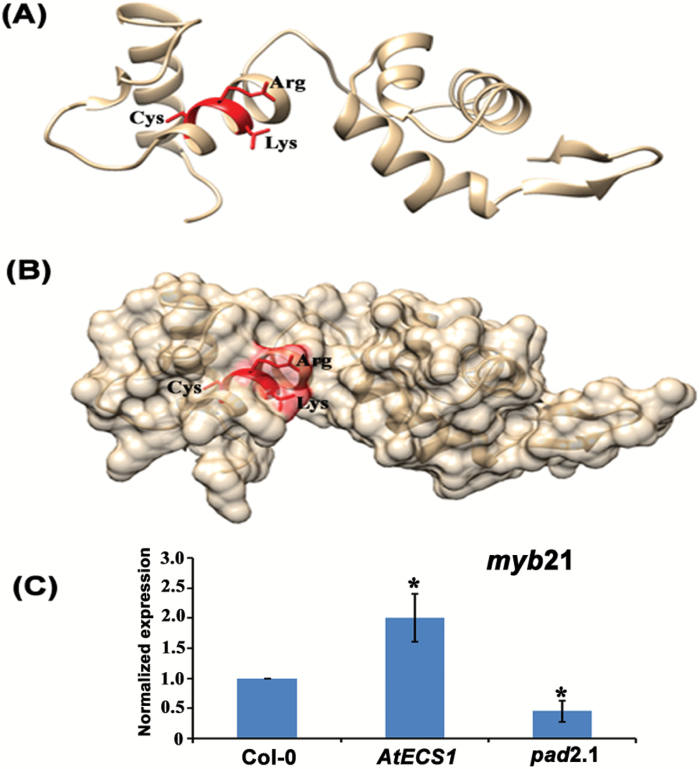
*In silico* prediction of the site for *S*-glutathionylation in MYB21 protein and effect of altered GSH on the expression of *myb21* gene. (A) Ribbon diagram of the homology model of MYB21 protein model with the amino acid residues required for GSH docking. (B) Molecular surface of MYB21 protein model with the amino acid residues required for GSH docking, clearly demonstrating that Cys residue was not located on the surface of the GSH binding pocket. Thus, there was little chance of *S*-glutathionylation of MYB21. (C) Quantitative RT-PCR revealed up-regulated and down-regulated expression of *myb21* in the *AtECS1* and *pad2.1* lines, respectively. Expression of *myb21* in *AtECS1* and *pad2.1* have been normalized with the expression of the same gene in Col-0. Data are presented as mean ±SE (*n*=3). ANOVA followed by Student–Newman–Keuls multiple comparison test was used (GraphPad InStat software, v. 3.1). *P<0.05, **P<0.01, ***P<0.001. (This figure is available in color at *JXB* online.)

*In silico* analysis of MYB21 revealed that there was a very low chance of its *S*-glutathionylation, as the cysteine residue was found to be embedded inside the protein’s globular structure (Fig. 6A, B). Quantitative RT-PCR revealed elevated expression of *myb21* in *AtECS1* and its lower expression in *pad2.1* in comparison with Col-0 (Fig. 6C). 

There are two ways by which GSH can regulate the expression of *BiP3* and *HSP70B* through MYB21. The first is by GSH activating MYB21 by post-translational modification (*S*-glutathionylation) after which its capacity to interact with and activate the promoters of *HSP70B* and *BiP3* is enhanced. The second is by enhanced expression of MYB21. The results indicated that GSH plays a key role in activating the promoters of *HSP*s by elevating the expression of *myb21.* Changes in GSH level in the plant cell also change the plant cell’s redox status, which also plays an important role in activating various proteins.

### Identification of other proteins regulated by MYB21 and BZIP10 in response to the elevated level of GSH

**Fig. 7.**
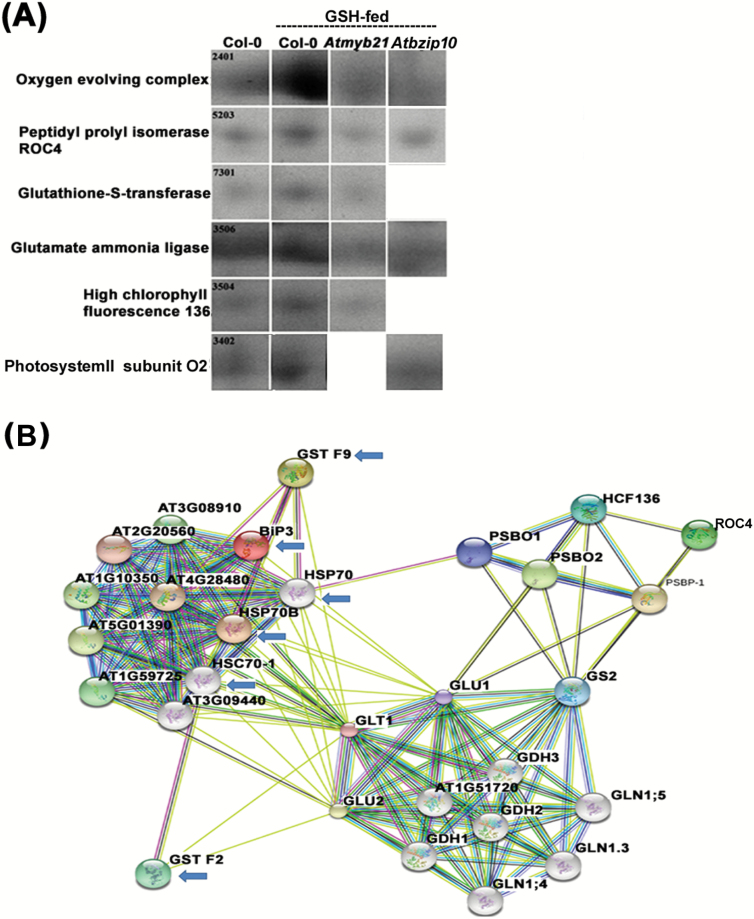
Identification of proteins whose expression is regulated by MYB21 and BZIP10 in response to GSH feeding. (A) Comparative proteomic analysis from the total proteins isolated from Col-0, GSH-fed Col-0, GSH-fed *Atmyb21*, and GSH-fed *Atbzip10* mutant revealed the proteins that were regulated by MYB21 and BZIP10 in response to GSH feeding. Each spot resembles a protein with standard spot number. See Supplementary Fig. S10 for 2DE gel picture of comparative proteomics analysis. (B) STRING 10 software protein interaction analysis of differentially expressed proteins with HSPs. Blue arrow depicts the interaction of GSTs with HSPs. (This figure is available in color at *JXB* online.)

**Fig. 8.**
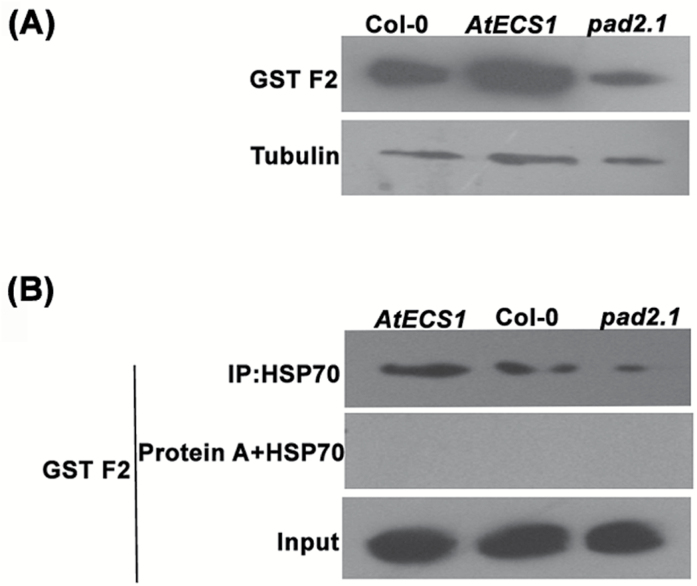
GST protein expression and interaction study with HSP70 under altered GSH condition. (A) Western blot analysis of GST F2 in three Arabidopsis lines, viz. wild type Col-0, transgenic *AtECS1*, and mutant *pad2.1*, revealed its up-regulation in *AtECS1* and down-regulation in *pad2.1.* (B) Protein co-immunoprecipitation followed by western blot demonstrated the elevated level of interaction of GST F2 with HSP70 in *AtECS1*. Input: loading control; protein A+HSP70: non-specific binding control.

Proteins regulated by MYB21 and BZIP10 in response to GSH were identified by using two-dimensional gel electrophoresis (2DE) followed by MALDI TOF/TOF tandem mass spectrometry (MS/MS) from the proteins isolated from Col-0, GSH-fed Col-0, *Atmyb21* and *Atbzip10* (see Supplementary Fig. S10). Overall mean coefficient of variation of differentially expressed proteins was 69.76 suggesting significant differences in protein expression. Total protein spots identified in Col-0, GSH-fed Col-0, *Atmyb21*, and *Atbzip10* were 490, 373, 250, and 378, respectively. There were 15 common spots that matched to every member. Among the differentially expressed proteins in the above-mentioned plant samples, glutathione-*S*-transferase, peptidylprolyl isomerase (PPIase) ROC4, high chlorophyll fluorescence 136, oxygen evolving protein, photosystem II subunit O-2 (PSBO2) and glutamate ammonia ligase GS2 were observed to be up-regulated in GSH-fed Col-0 and the same proteins were observed to be down-regulated in GSH-fed TF mutant lines (Fig. 7A; Table 1). STRING 10 software analysis of differentially expressed proteins revealed a strong interaction between HSP70 and glutathione-*S*-transferase (GST) in response to the elevated level of GSH (Fig. 7B). Amongst the above-mentioned proteins the expression of GST F2 was validated by western blot analysis (Fig. 8A). Together, the above results helped in the identification of important proteins other than HSPs that were regulated by MYB21 and BZIP10 in response to GSH feeding.

**Table 1.** MALDI TOF/TOF MS/MS-based identification of proteins whose expression is regulated by MYB21 and BZIP10 under an elevated GSH level

SSP no.^*a*^	Experimental *M*_r_/pI^*b*^	Average fold change (*Atmyb2 1*/*Atbzip 10*)^*c*^	Protein	Accession no.^*d*^	Mascot score^*e*^	Plant
2401	33.00/5.66	0.60/0.79	Oxygen-evolving protein	gi|22571	84	Arabidopsis
5203	28.00/8.83	0.17/0.69	Peptidylprolyl isomerase ROC4	gi|21555831	39	Arabidopsis
7301	24.00/6.28	0.68/>1.0	Glutathione-*S*-transferase F2	gi|21555418	70	Arabidopsis
3506	20.00/5.57	0.80/0.41	Glutamate ammonia ligase	gi|62319277	176	Arabidopsis
3504	44.00/6.70	0.01/>1.0	High chlorophyll fluorescence 136	gi|15237225	50	Arabidopsis
3402	35.00/5.91	>1.0/0.29	Photosystem II subunit O-2	gi|15230324	86	Arabidopsis

^*a*^ Identified differentially accumulated proteins in Col-0, GSH-fed Col-0, and GSH-fed *Atmyb21* and *Atbzip10* mutants.

^*b*^ Mass of protein in kDa and pI.

^*c*^ Intensity fold change.

^*d*^ NCBI accession number of identified protein spots.

^*e*^ Statistical probability of true positive identification of predicted proteins calculated by MASCOT (http://www.matrixscience.com).

### GSH has a role in increasing the GST interaction with HSP70

**Fig. 9.**
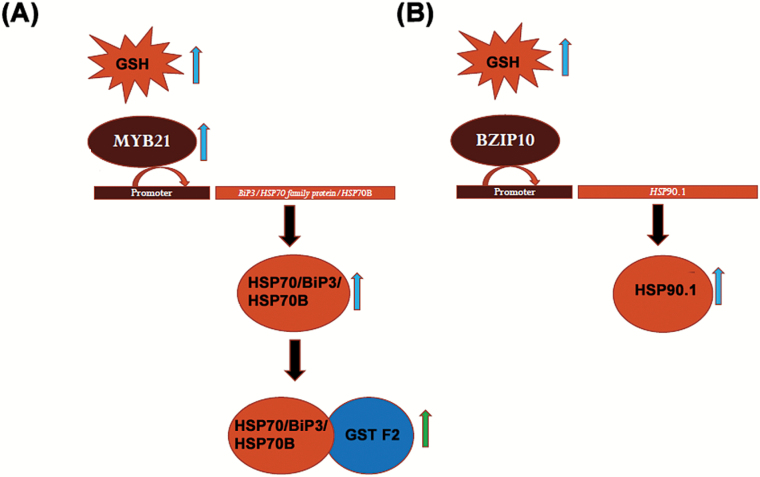
A suggested working model for GSH and *HSP* interplay. (A) Elevated GSH level in plant cell induces the expression of *myb21* TF, which activates the promoters of HSP70 family genes, i.e. *BiP3* and *HSP70B*, and up-regulates their expression. GSH also plays important role in inducing *GST F2* gene through MYB21. Downstream GSH also modulates the chaperonic effect of HSP70 by elevating its interaction with GST F2. (B) GSH also up-regulates the expression of HSP90.1 at both gene and protein level by activating its promoter through BZIP10. Blue arrow, up-regulation of genes and proteins; green arrow, elevated protein interaction. (This figure is available in color at *JXB* online.)

Total proteins, isolated from *AtECS1*, *pad2.1*, and Col-0, were co-immunoprecipitated with rabbit polyclonal anti-HSP70 primary antibody. Western blot was performed using co-immunoprecipitated eluted sample of total protein and rabbit polyclonal anti-HSP70 primary antibody with rabbit polyclonal anti-GST phi primary antibody. The results revealed highest expression of GST in co-immunoprecipitated eluted sample of *AtECS1* and least expression in an eluted sample of *pad2.1* (Fig. 8B). The results cumulatively demonstrated the key role of GSH not only in inducing GST biosynthesis but also in increasing the chaperonic effect of HSP70 towards GST. A proposed model for the regulation of *HSP*s through GSH is shown in Fig. 9.

## Discussion

Various environmental stresses have an adverse effect on plant metabolism, cellular homeostasis, and major physiological processes, as is well-established and the subject of many studies. The important role of GSH in the development of stress tolerance in plants has been widely recognized (Dron *et al.*, 1988; Wingate *et al.*, 1988; Tausz *et al.*, 2004; Ball *et al.*, 2004; Noctor *et al.*, 2012). Our previous investigations also pointed toward the importance of GSH in inducing various stress-related genes and proteins, especially HSPs, to combat both biotic and abiotic stress conditions *in planta* (Ghanta *et al.* 2011, 2014; Sinha *et al.* 2015; Datta *et al.*, 2015; Kumar *et al.*, 2015, 2016). In this investigation, we probed the mechanism through which GSH regulates important stress modulators, viz. the *HSP*s *BiP3*, *HSP70B*, and *HSP90.1.* This study demonstrated the proteins that are induced by TFs such as MYB21 and BZIP10 in response to the elevated level of GSH. Our study also demonstrated the functional role of GSH in changing the interaction efficiency of HSP70 towards GST.

Most of the stress- and defense-related genes and proteins, especially *HSP*s such as *BiP3*, *HSP70B*, and *HSP90.1*, were found to be down-regulated under altered GSH conditions in Arabidopsis GSH mutant *pad2.1*, whereas, interestingly, the same genes were found to be up-regulated in GSH-fed wild type Arabidopsis (Ghanta *et al.*, 2011; Sinha *et al.*, 2015; Kumar *et al.*, 2016). Heat stress also has a role in the accumulation of GSH in the frost-sensitive wheat plant (Kocsy *et al.*, 2004), and also it has been reported that in response to heat stress, *HSP70B* was induced (Sung *et al.*, 2001*a*), which may suggest a relation between levels of GSH and *HSP70B*. The contribution of HSP90.1 in mitigating environmental stress through developing RPS2-mediated disease resistance is an established fact (Krishna and Gloor, 2001; Takahashi *et al.*, 2003). BiP3 or HSP70 family proteins have also been reported to play an important role in mitigating both abiotic and biotic stress in *Nicotiana* and soybean (Alvim *et al.*, 2001; Wang *et al.*, 2005; Valente *et al.*, 2009). Elevated GSH levels in eukaryotic cells produce a reducing environment (Deponte, 2013) and this reducing environment could also induce these stress- and defense-related *HSP*s. Therefore, we determined the expression of these *HSP*s in β-ME- and DTT-treated Col-0 to mimic reducing conditions and observed insignificant changes in the expression of these *HSP*s in comparison with untreated Col-0. Previous studies of our lab reported a 2.24-fold higher yield of GSH in the *AtECS1* transgenic line, overexpressing γ-ECS, the key enzyme in GSH biosynthesis, in comparison with Col-0. In GSH-fed Col-0, a more than 2.0-fold increase in GSH content has been recorded as well. On the other hand, in BSO-fed Col-0 and *pad2.1*, the GSH content was reduced by about 5.5- and 3.0-fold, respectively (Datta *et al.*, 2015; Kumar *et al.*, 2015). This result clearly indicated that the content of GSH has a key role in inducing these *HSP*s. In low-GSH conditions (BSO-treated Col-0 or *pad2.1*), the *HSP* expression is not as much inhibited by far, as expected from the very low (~10%) GSH levels obtained. This may be due to a basal level of GSH in the BSO-treated cells. This basal level of GSH can also play a role in the regulation of *HSP* expression, but is unable to exhibit the enhanced expression of *HSP*s. Similarly, in *pad2.1* a basal level of GSH remains in the plant cell that can also regulate the expression of *HSP*s.

Induction of *BiP3*, *HSP70B*, and *HSP90.1* through GSH can occur in several possible ways. For instance, GSH can induce expression of *HSP*s at transcript level via activation of their promoters. A second possible way, in response to stress, is via the accumulation of GSH in plant cells, which changes the cellular redox homeostasis and can result in the induction of these *HSP*s. Post-translational modification of these HSPs by *S*-glutathionylation is a third way to regulate the proteins’ stability, and a fourth possible way is by modulating the stability of their mRNA.

To obtain further in-depth insight, a promoter activation assay on Arabidopsis protoplasts was performed and the results revealed that the elevated GSH level induced the expression of *HSP*s by activating their promoters. It is obvious that GSH itself cannot bind to the promoter sequences of these *HSP*s to activate them. Consequently, the next task was to identify the transcriptional regulators that activate the promoters of these *HSP*s in response to GSH. Previous reports have indicated that various *HSP*s that had MYB binding site elements in their upstream region were also found to be induced in response to drought stress (Reddy *et al.*, 2014). Relocalization of the TF complex UBL-5–DVE-1 and bZIP to the nucleus activated the expression of the chaperones HSP6 (mtHSP70) and HSP60 (mt chaperonin) (Kirstein-Miles and Morimoto, 2010). Our transcript analysis of the TF mutant lines showed no marginal change in the induction of *BiP3* and *HSP70B* in the GSH-fed *Atmyb21* mutant and of *HSP90.1* in the GSH-fed *Atbzip10* mutant line in comparison with GSH-fed Col-0. This observation was confirmed by a co-transfection assay of the *BiP3* and *HSP70B* promoters with *myb21* in *Atmyb21* protoplasts and *HSP90.1* promoter with *bzip10* in *Atbzip10* mutant protoplasts. Previous reports also demonstrated that GSH is an important factor in determining the basal JA-responsive gene expression and suggested that a GSH-dependent control point regulates JA signaling (Han *et al.*, 2013). The contribution of BZIP10 to the oxidative stress response in Arabidopsis is known (Kaminaka *et al.*, 2006). Homology modelling of MYB21 showed no site for glutathionylation, and transcript analysis revealed the up-regulation of *myb21* in a GSH overexpression line. Altogether, it is evident that in response to stress the cellular GSH content increases, which induces expression of *myb21* and ultimately activates the promoters of *BiP3* and *HSP70B*.

Results of comparative proteomics analysis on wild type Col-0 and TF mutant plants revealed other proteins, such as GST, PPIase ROC4, high chlorophyll fluorescence 136 (HCF 136), oxygen evolving protein, photosystem II subunit O-2 (PSBO2) and glutamate ammonia ligase, that were also regulated by MYB21 and BZIP10 in response to an elevated level of GSH. The function of GSH in enhancing the expression and activity of GST is an established fact (Mauch and Dudler, 1993; Loyall *et al.*, 2000; Sinha *et al.*, 2015). Down-regulation of PPIase ROC4 in stress-treated *pad2.1* at both gene and protein levels shows a role of GSH in inducing this gene (Kumar *et al.*, 2015). Previous investigation also suggested the importance of accumulation of glutamate ammonia ligase in the increased assimilation of ammonium in response to salt stress in potato (Teixeira and Fidalgo, 2009). These observations suggest a role of GSH in inducing these stress- and defense-related proteins along with HSPs through the TFs MYB21 and BZIP10 in response to environmental stress.

STRING 10 protein–protein interaction analysis pointed towards an interaction between HSP70 family proteins and other differentially expressed proteins in GSH-fed Col-0, *Atmyb21*, and *Atbzip10* mutants. Among the differentially expressed proteins, GST showed maximum chance of interacting with HSP70 followed by PPIase ROC4, GS2, HCF, and PSBO2. HSP70 induced by stress plays an important role in mitigating the aggregation of stress-denatured proteins and helps in the refolding of non-native proteins for restoring their biological function through iterative cycles of adenine nucleotide hydrolysis-dependent peptide binding and release (De Maio, 1999; Sung *et al.*, 2001*b*; Wang *et al.*, 2004). A protein co-immunoprecipitation assay followed by western blot analysis revealed an enhanced level of interaction of GST with HSP70 in response to an elevated level of GSH in plant cells. Previous reports also pointed towards the higher affinity of HSP70 for GST under GSH-rich conditions (Robin *et al.*, 2003). These observations suggest that GSH not only plays an important part in the induction of GST, but also modulates its interaction with HSP70 chaperone. GSTs are important in combating a wide range of abiotic and biotic stresses (Roxas *et al.*, 1997; Dalton *et al.*, 2009). In response to stress conditions, the chances of denaturation of stress-mitigating proteins such as GST increases. At that point different HSPs can interact with GST and act as a molecular chaperone to form an HSP–GST complex that can help in the restoration of function under stress conditions by maintaining its active form.

In this study, we have revealed a fascinating interplay of GSH and HSPs that execute important functions in combating environmental stress in the plant system. The expression levels of *HSP*s were positively regulated by GSH. We further suggest that GSH induces *BiP3* and *HSP70B* by up-regulating the expression of *myb21* (Fig 9A), which plays a vital role in activating the promoters of both *BiP3* and *HSP70B*. On the other hand, BZIP10 elevates the expression of *HSP90.1* by activating its promoter in response to an increased level of GSH (Fig. 9B). We also observed an increased level of interaction of GST F2 with HSP70, which pointed towards its greater chaperone-like effect in response to the elevated level of GSH in plant cells (Fig. 9A). Together, our study has revealed the molecular mechanism through which GSH modulates the expression and chaperone-like effect of HSPs. However, the possibility of an indirect regulation of the expression of HSPs brought upon by MYB21/BZIP10 cannot be ruled out assuming that these TFs can interact with or are modulated by several other entities. This may be another appealing area of investigation in the near future that will improve our understanding of the contributions of HSPs in stress tolerance and management. Furthermore, it will be interesting to explore the effect of interactions of different proteins with HSPs to mitigate stress.

## Supplementary data

Supplementary data are available at *JXB* online.

Fig. S1. Transcript study of HSP genes in Col-0, *AtECS1*, *pad2.1*, and GSH-fed and BSO-fed Col-0 with tubulin as a reference gene.

Fig. S2. Transcript study of HSP genes in altered GSH condition.

Fig. S3. Western blot analysis of HSP70 and HSP90.1 protein expression in Col-0, *AtECS1*, *pad2.1*, and GSH-fed and BSO-fed Col-0.

Fig. S4. Promoter activation analysis of *proBiP3*, *proHSP70B*, and *proHSP90.1* in response to altered GSH conditions.

Fig. S5. GFP fluorescence analysis through ImageJ/Fiji software.

Fig. S6. Promoter sequences of *HSP*s.

Fig. S7. Western blot analysis of HSP expression in Col-0, GSH-fed Col-0, *Atmyb21*, GSH-fed *Atmyb21*, *Atbzip10*, and GSH-fed *Atbzip10*.

Fig. S8. Effect on the expression of *HSP*s in GSH-fed *AtAP2* and *Atein3* mutant lines.

Fig. S9. Co-transfection of the protoplasts *Atmyb21* in GSH-fed condition.

Fig. S10. 2DE gel picture of comparative proteomics analysis of Col-0, GSH-fed Col-0, GSH fed *Atmyb21*, and GSH-fed *Atbzip10* mutants.

Table S1. List of primers used in the quantitative RT-PCR.

Table S2. Value of GFP fluorescence in the protoplasts of transfected plant samples with altered GSH condition.

Supplementary Tables Figure

## Abbreviations:

BSO: buthionine sulphoximine
2DE: two-dimensional gel electrophoresis
DsRed: *Discosoma*-specific red fluorescent protein
DTT: dithiothreitol
GFP: green fluorescent protein
GSH: glutathione
GST: glutathione-*S*-transferase
HSP: heat shock protein
β-ME: β-mercaptoethanol
TF: transcription factor
YFP: yellow fluorescent protein.
