# Supplementary material for: Glutathione modulates the expression of heat shock proteins via the transcription factors BZIP10 and MYB21 in Arabidopsis
Source: J Exp Bot. 2018 May 2;69(15):3729–43. doi: 10.1093/jxb/ery166 (PMC6022672; doi:10.1093/jxb/ery166)
Supplement: Supplementary Tables Figure [file ery166_suppl_supplementary_tables_figure.pdf]

Supplementary Data

Glutathione modulates transcription of heat shock proteins in response to stress via BZIP10 and MYB21 transcription factors in *Arabidopsis thaliana*

Deepak Kumar and Sharmila Chattopadhyay*

Fig. S1. Transcript study of HSPs gene in Col-0, *AtECS1*, *pad2.1*, GSH fed, BSO fed Col-0 by taking tubulin as a reference gene.

Fig. S2. Transcript study of *HSPs* gene in altered GSH condition.

Fig. S3. Western blot analysis of the HSP70 and HSP90.1 protein expression in Col-0, *AtECS1*, *pad2.1*, GSH fed and BSO fed Col-0.

Fig. S4. Promoter activation analysis of *proBiP3*, *proHSP70B* and *proHSP90.1* in response to altered GSH conditions.

Fig. S5. GFP fluorescence analysis through ImageJ/Fiji software.

Fig. S6. Promoter sequences of *HSPs*.

Fig. S7. Western blot analysis of HSPs protein expression in Col-0, GSH fed, *Atmyb21*, GSH fed *myb21*, *Atbzip10* and GSH fed *Atbzip10*.

Fig. S8. Effect on the expression of *HSPs* in GSH fed *AtAP2* and *Atein3* mutant line.

Fig. S9. Co-transfection of the protoplasts *Atmyb21* in GSH fed condition.

Fig. S10. 2DE gel picture of comparative proteomic analysis of Col-0, GSH fed Col-0 GSH fed *Atmyb21* and GSH fed *Atbzip10* mutants.

Table S1. List of primers used in the quantitative RT-PCR.

Table S2: Value of GFP fluorescence in the protoplasts of transfected plant samples with altered GSH condition.

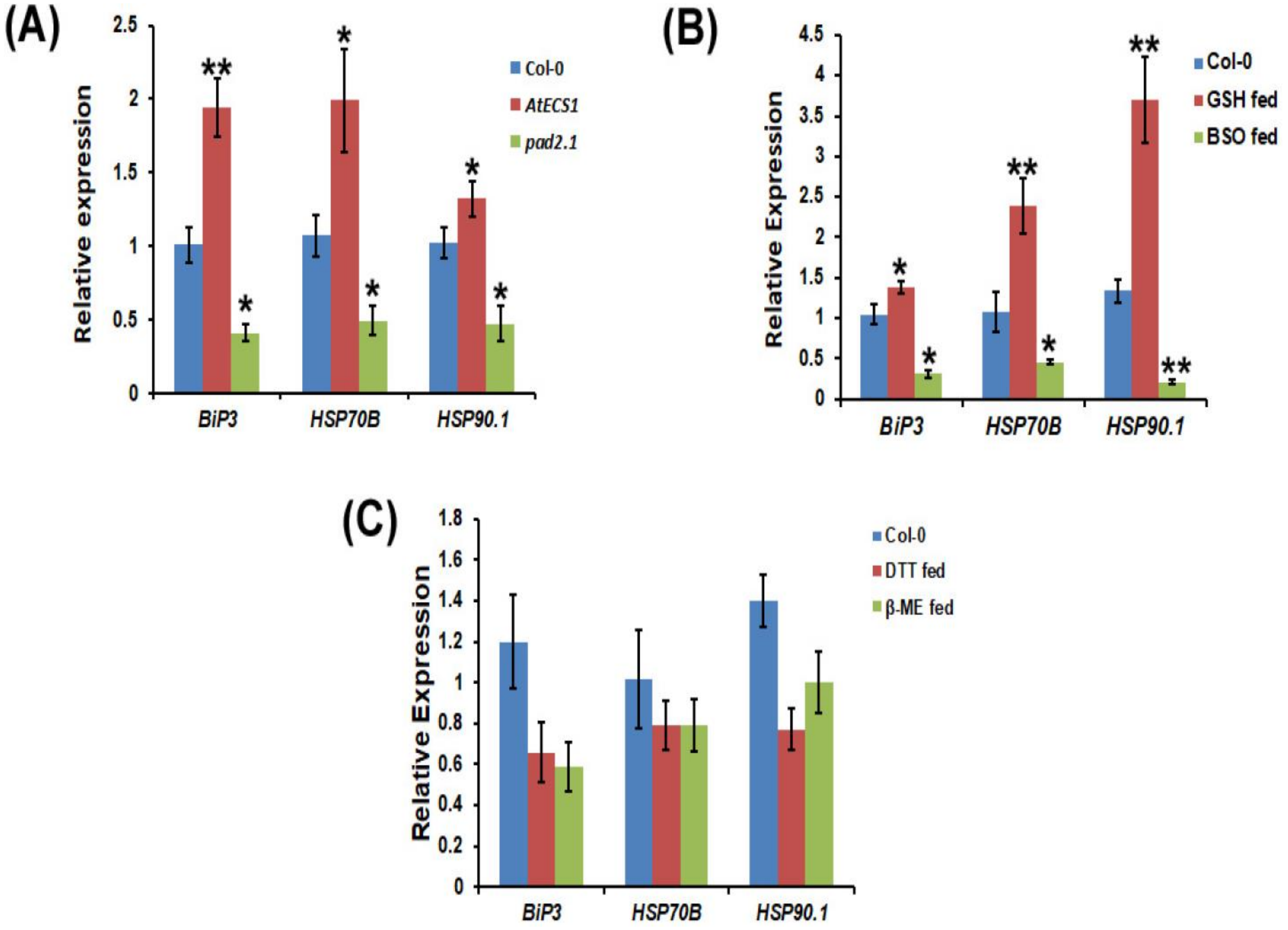

Supplementary Figure S1. Transcript study of HSPs gene in Col-0, *AtECS1*, *pad2.1*, GSH fed, BSO fed Col-0, DTT and β-ME fed Col-0 by taking tubulin as a reference gene. Data are presented as mean± SE (*n*=3). ***P*<0.05.

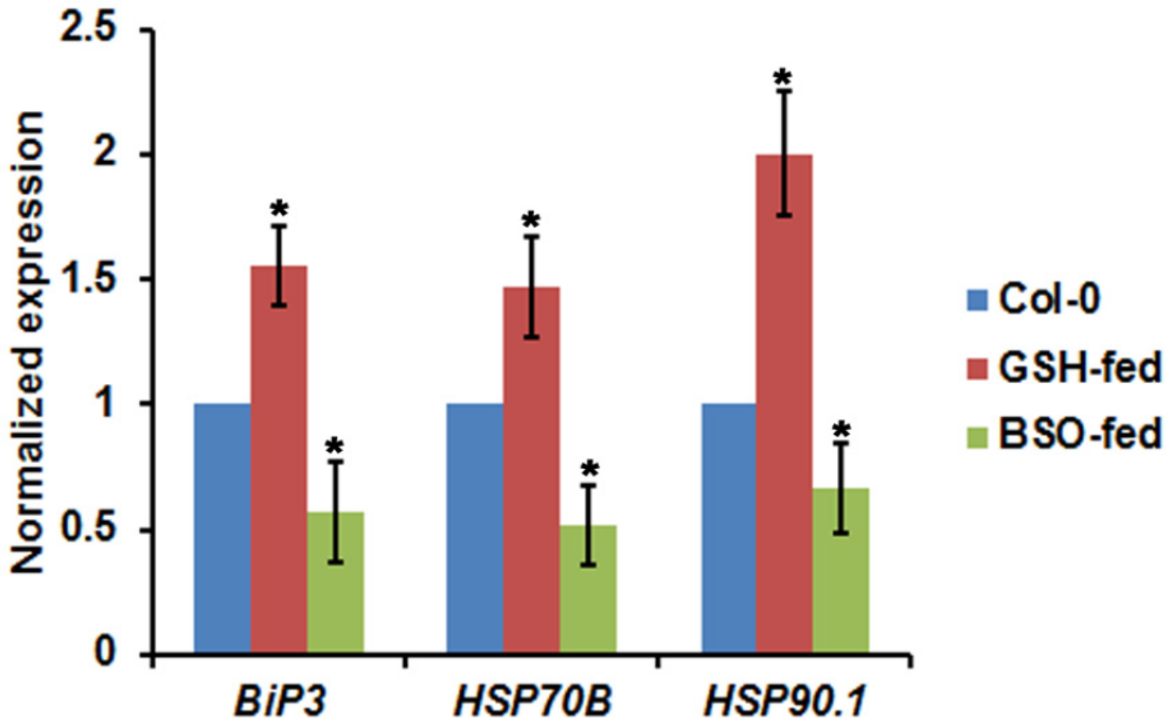

Supplementary Figure S2. Transcript study of *HSPs* gene in altered GSH condition. Quantitative RT-PCR analysis revealed up-regulation of *BiP3*, *HSP70B* and *HSP90.1* in GSH fed Col-0 and down-regulation of the same gene in BSO fed Col-0. ***P*<0.05.

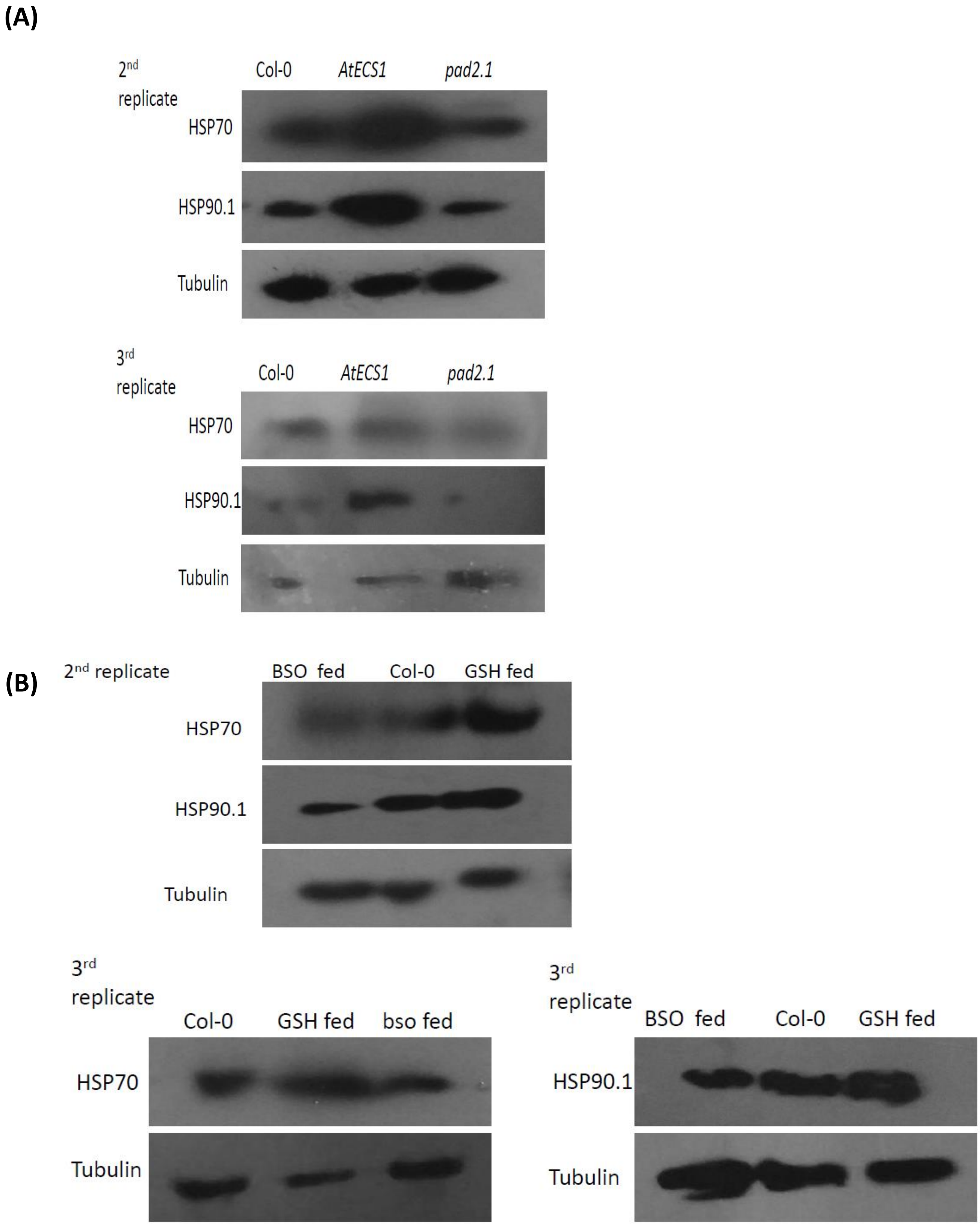

Supplementary Figure S3. Western blot analysis of the HSP70 and HSP90.1 protein expression in Col-0, *AtECS1*, *pad2.1*, GSH fed and BSO fed Col-0.Replicates of western blotting for the results mentioned in (A) Fig. 1D and (B) Fig. 1E.

Supplementary Figure S4. Promoter activation analysis of *proBiP3*, *proHSP70B* and *proHSP90.1* in response to altered GSH conditions. Minimum 10 protoplasts have been taken for GFP fluorescence analysis through ImageJ/Fiji software.

(A)

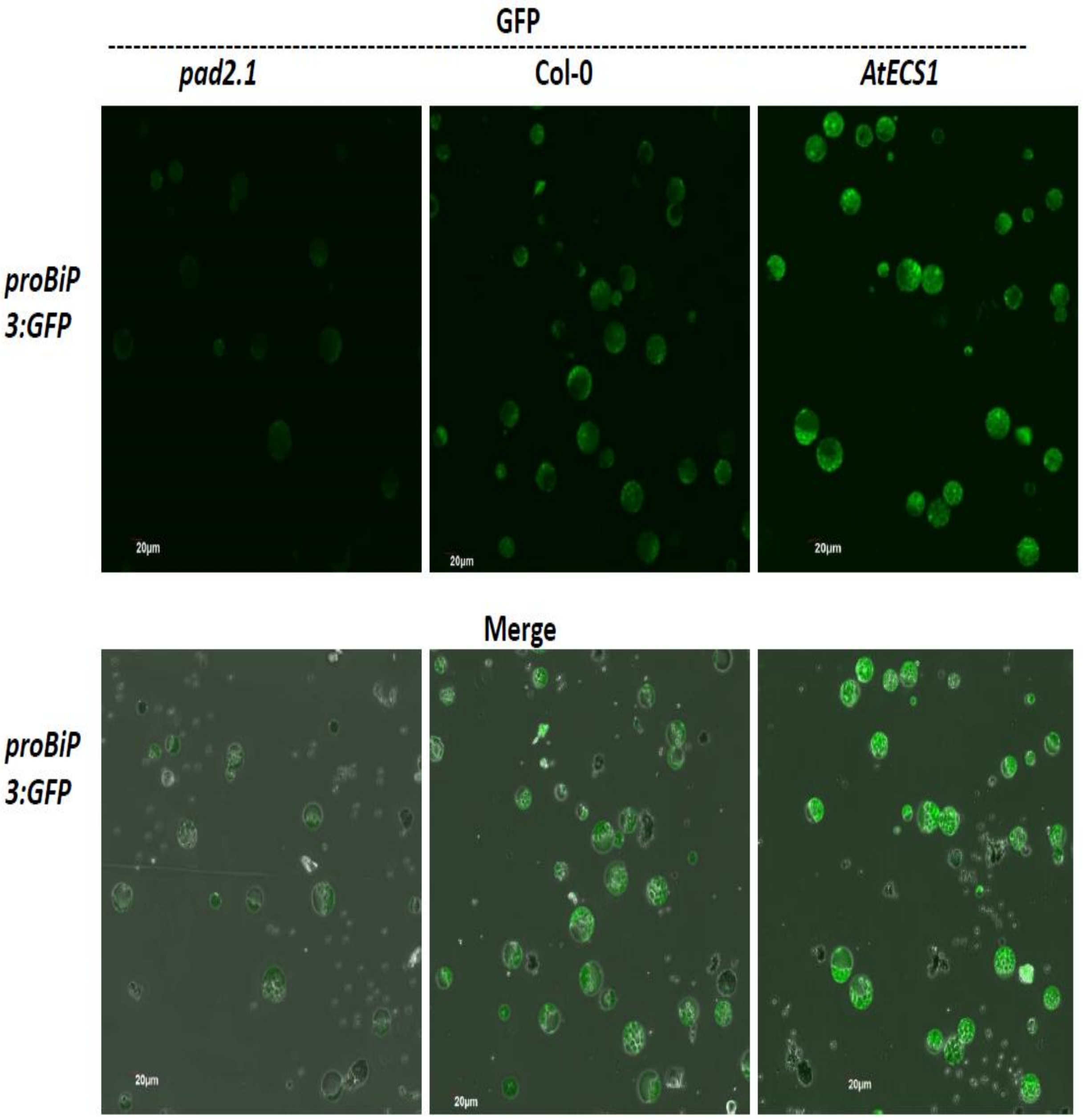

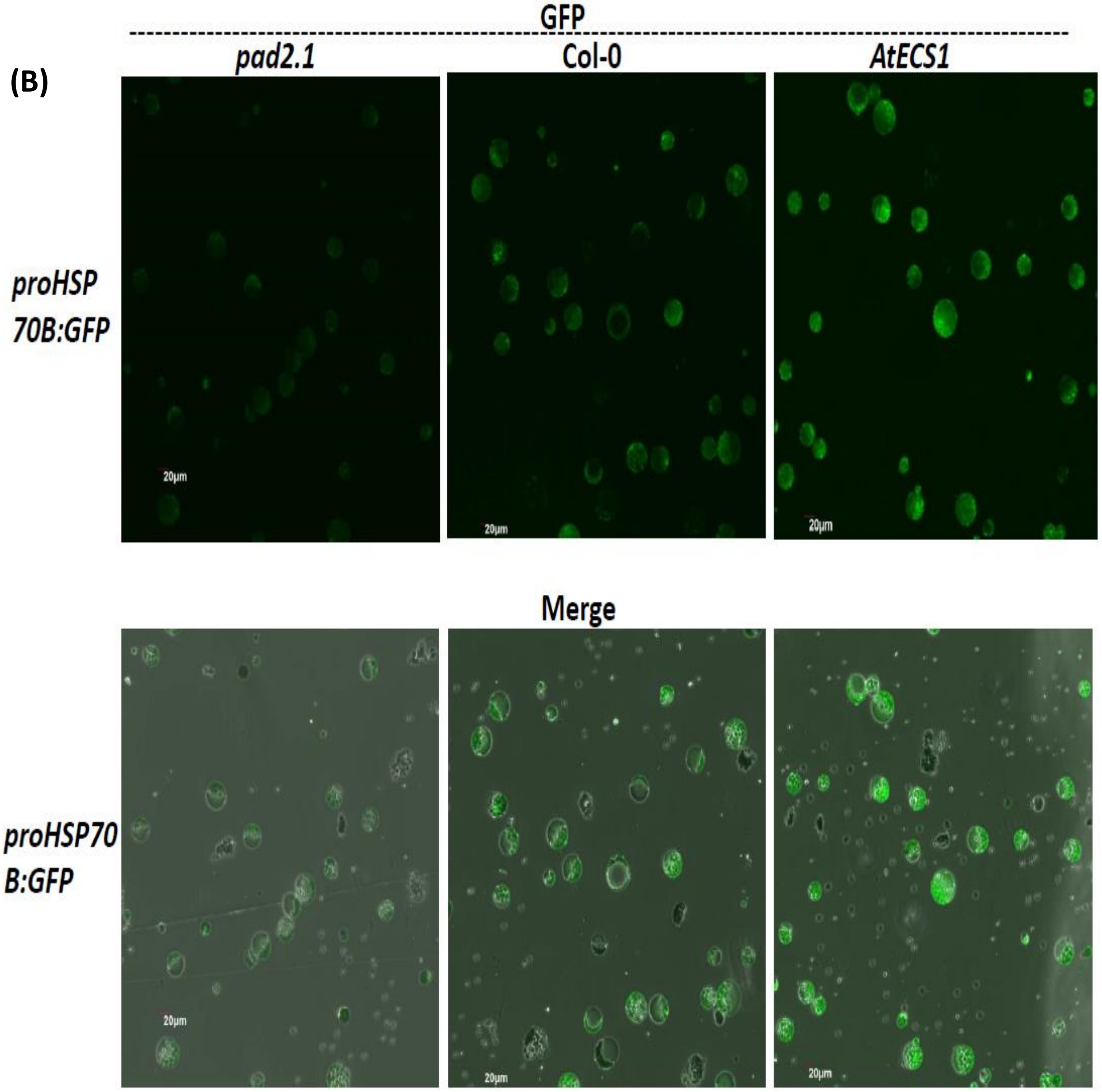
GFP
(B)
pad2.1
Col-0
AtECS1
proHSP
70B:GFP
20µm
20µm
20µm
Merge
proHSP70
B:GFP
20µm
20µm
20µm

(C)

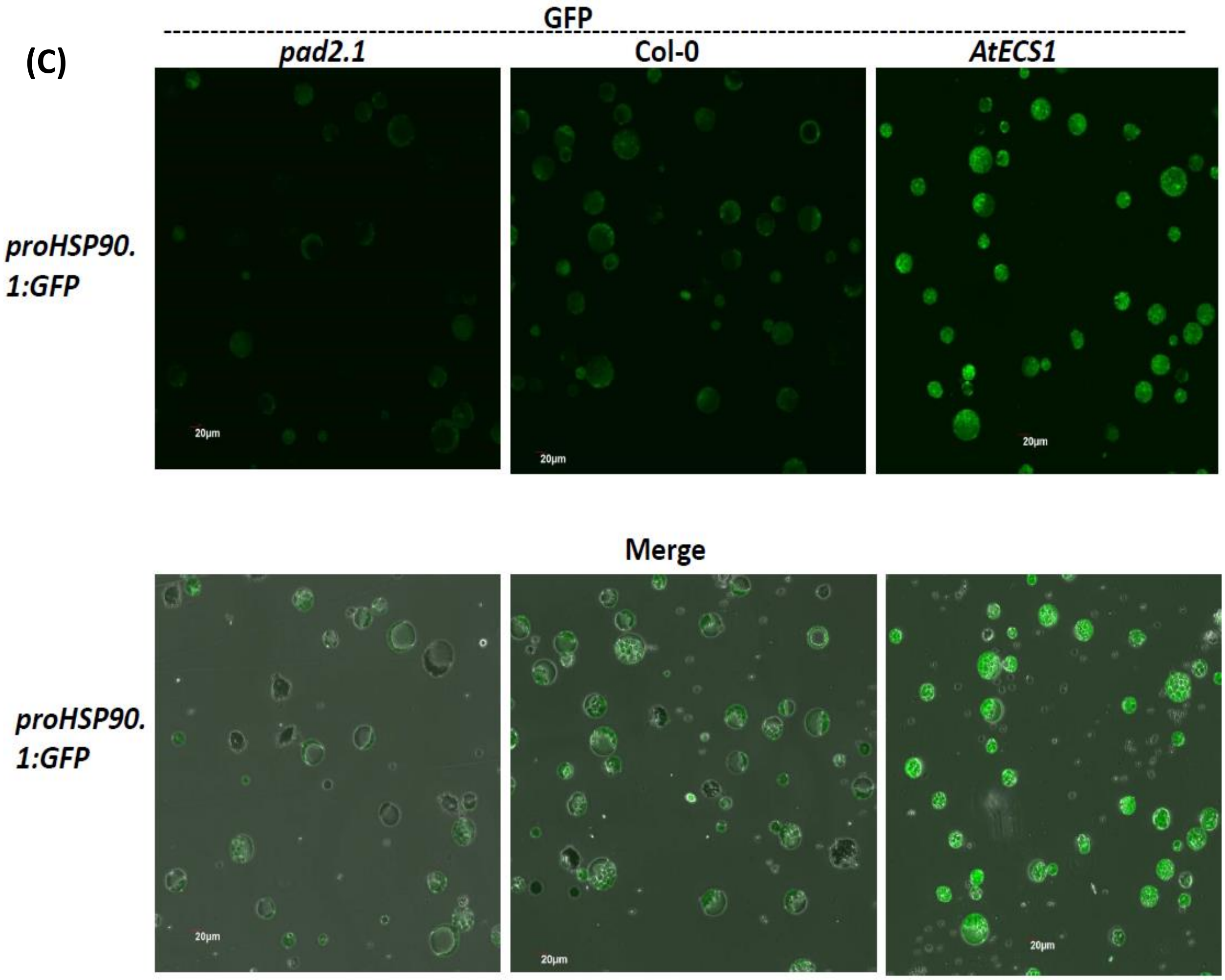
GFP
pad2.1
Col-0
AtECS1
proHSP90.
1:GFP
20μm
20μm
20μm
Merge
proHSP90.
1:GFP
20μm
20μm
20μm

(D)

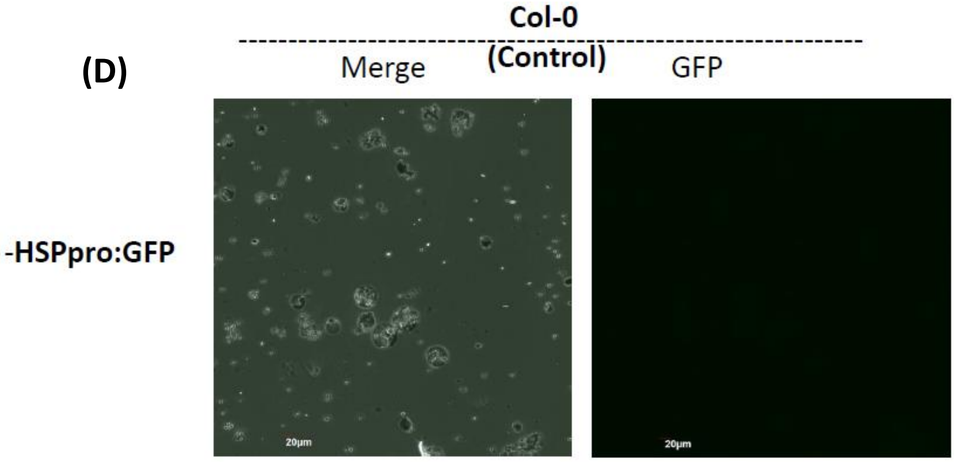
Col-0
(Control)
Merge
GFP
-HSPpro:GFP
20μm
20μm

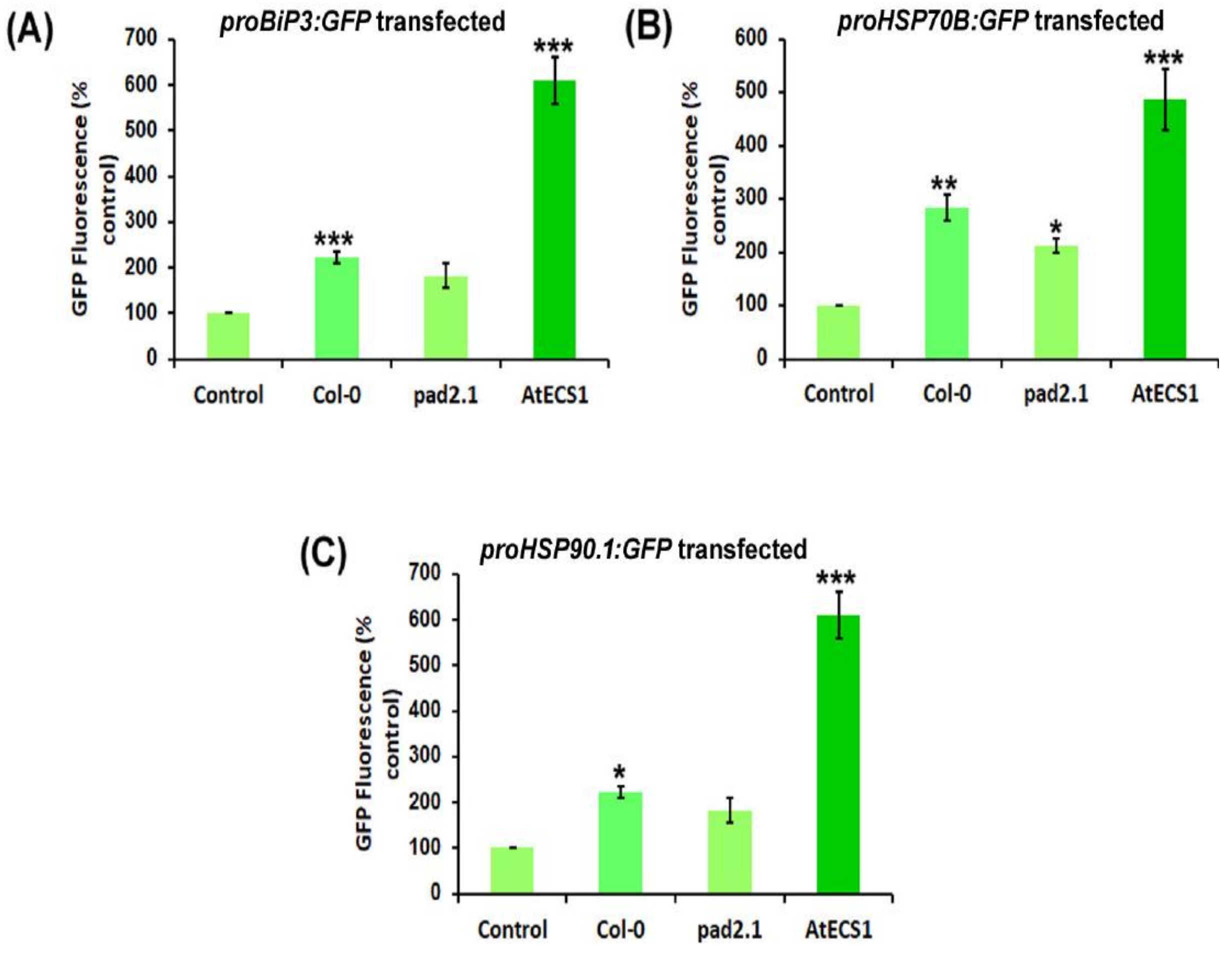

Supplementary Figure S5. GFP fluorescence analysis through ImageJ/Fiji software in (A) *BiP3* (B) *HSP70B* and (C) *HSP90.1 promoter construct transfected protoplast of Col-0, pad2.1 and AtECS1.* Data are presented as mean± SE ($n \geq 10$) *****$P < 0.05$.

Promoter site

HSP70B **(TFs binding sites on HSP70B PROMOTER)**

GGCTTTTCCTTTCTCGTCTTCGAAATCACAGAACAAGTGAAGAAAGAAGACGTAAAC
AAAATATTGAAAATCCTCCAGAACTTACACTGGGCCTTTTATTCTATATACGGGCCT
ACAAGTTTATACCATATGGGCTTTAATAGGCCCATTTAATTATCAAGCGGTCGCCG
GAGATAAAATATATCCCGGTCGGTGAATCCAGAACTCTCTTGTACGTTTGCGCG
ATTTCTCCACCTTTCCACAATCCCCTGGGTTGTGCCACGACCTTTTTTCTCGAAAT
GTCTCGTTCCTCTCGTCGGATTCGTATATATAGCTTCTTCCATCGTTTCCGATTCTT
CATCAAACAGATAAACAAACAAAAGAAATCGAAAAACCTCACTTCCAATTTCATTCAA
TTACTGAAGCTTTTTTTTAGCAACA

HSP90.1 **(TFs binding sites on HSP90.1 PROMOTER)**

CGTACGTTTTTCAAAGATCGTGTATTTTGGATTTTGGTTCCAGTTCCAGTTCCAGT
TCCAGTTCCAGACTTAACGTAGACGACAACCTAGGGTTCAAAGGCAATTCATTCAT
ACCTAATGGGCCGAATGAATTCATGGGCCATGTTAGAGGCAACTAAAAGGCTGGAC
GAAACTATCTATTTTAGCCTTTTAGGGGAGGTAACGTATTAATGTCCCCAACTATCTT
TTATAGGATGCATAGTTGGTTTTAACTAACATTAAAGGTGTTACTAACAATGGGCAAT
GATGGTAAAATTATAACGCAGTGGACACAATATAACTTAGCCATACCAATGCAAAGAA
TCTTGAAACTAGTATTAAACTCAGTCACGTATATGCTCTCCAGTCTATGCTTCGAA
GAGCTTTCTTCTAATCCCATACGAAGAAGCTCTCTATGTTTTTCTAATTTAATCAGGCT
TAGACAACTAGTTTCAAAGTCATAATTCTTGGTGTCTGAGTTGTCTTCCCTTTCCATCG
AGTTTTTTAAAGATCGTGTCTTTTGGTTTCCAGTTCCAGACTACGTAGGCGACACT
TCAGGGTTTCAAAGGCAATGGCACTAAAAGTCACTAATGGGCCTAACGACTTAATG
GGCCTTGTGAAACGTTACCCAATAAGAGACCTGGTTTCTCACTGAGTTCAACCATCT
TCTTCCCC

BiP3 **(TFs binding sites on HSP70 PROMOTER)**

TCACTACAATTACATTATTCACGCTGTTTTGTATACTATGTAATTTATATAATGTCAA
AAAAGACGGATTATAATGCCCATATTACCCTTCTAACAAAAAATAAAATAAAATAATAATA
TCCATATTACCCTTTGACAATTTCGAGTATACCATATGAATTATAACTTAGTATTAAAATA
TTGATTTACTCCAACGACATATTTTAAAAATCAAATGATATATGGTCAATTATTTAGTTT
TGAAAGTGAATTAATATTAGAAGCCAAAAACCTAGGGCCGCCATTAGGGTTGGTTT
GCCTGTTTTGTTGTTATCCAATATCCAATTCTTATTGTGCATTGATTCCAATACAAATA
CAACACTGAGAGTAATTAGAAAATGGCATATTTCTTATATATATGTCTGCGTGATAAATA
AATGGAGTGGTTAAGCATTAGTCATGTTCTTCCAAATATTACACCTCACCGCATATG
CAGTAGTTTTGGCATTACACTAATTCATTAATAACAAAAGAAATTATCCTTATGTAAA
TTATGACGCAAATCTTTTTATTTTATTAATCGGTACACGAAAAAAATCTTTTAATAATTCA
TGCATGTGATAAAAACATTGTTAATAATTTAATTTTGATTATAATGATAATTGTTTTGGA
TAACTATACATACATACGAAGAAGTCGTAAAACCTTTGAAAGATTTTGCGATGAC
GATTTAATGTCACGTGTCTGCTTGTGATTGGGTACTTTACGTGTGTAAAAAGTAAGG
AGCGCGCCAACACAAAATAACCCATTAAGCTTACGTGTCAAGAAGTGATTGGAGAG
GACACTCTACCGAGGCTAAATACGAATCATACTGAAGCACATATAAATAGACGACGA
ACTTCATACTCTTCCAAATAAACTAACAAACGAGATCGAAGAAGAGTTCTCAACAACG
AAAA

Supplementary Figure S6. Promoter sequences of *HSPs*. (A), (B) and (C) are the promoter sequences of *HSP70B, HSP90.1* and *BiP3.* Blue, violet, red and green letters denote the binding sequences for BZIP10, EIN3, MYB21 and APETALA2 transcription factors. TFs binding sequences were analysed by PlantPan sortware.

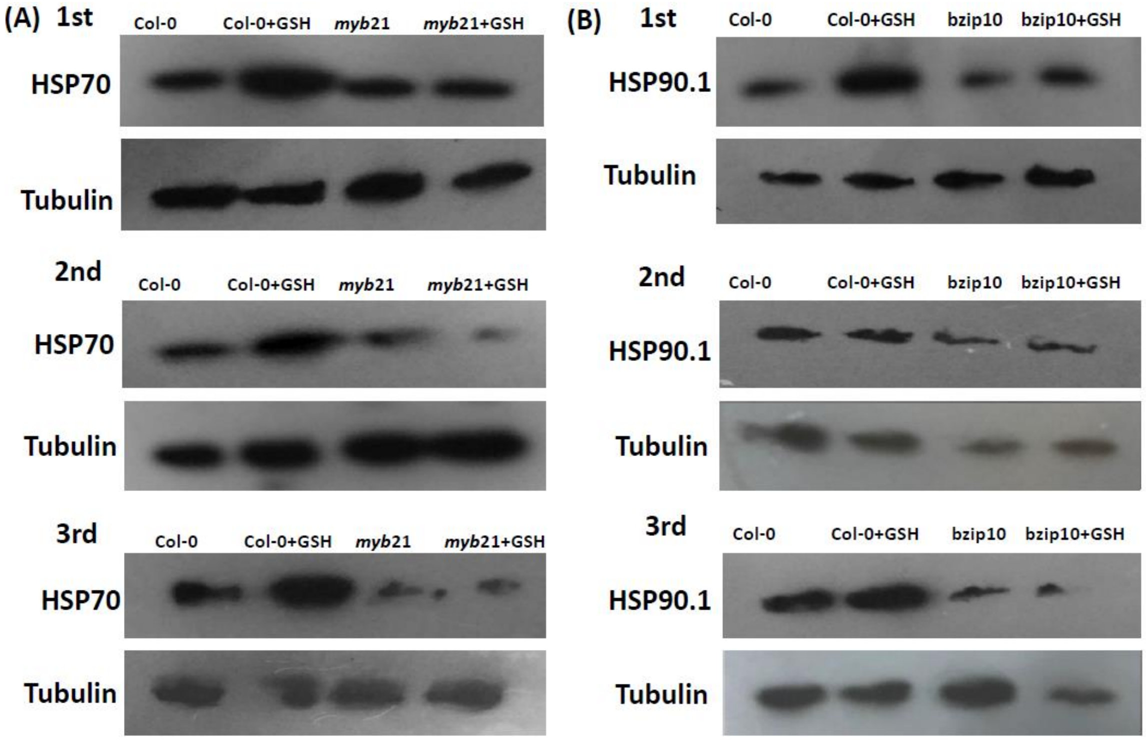

Supplementary Figure S7. Western blot analysis of HSPs protein expression in Col-0, GSH fed, *Atmyb21*, GSH fed *myb21*, *Atbzip10* and GSH fed *Atbzip10*. Replicates of western blotting for the results mentioned in (A) Fig. 1G and (B) Fig. 1H.

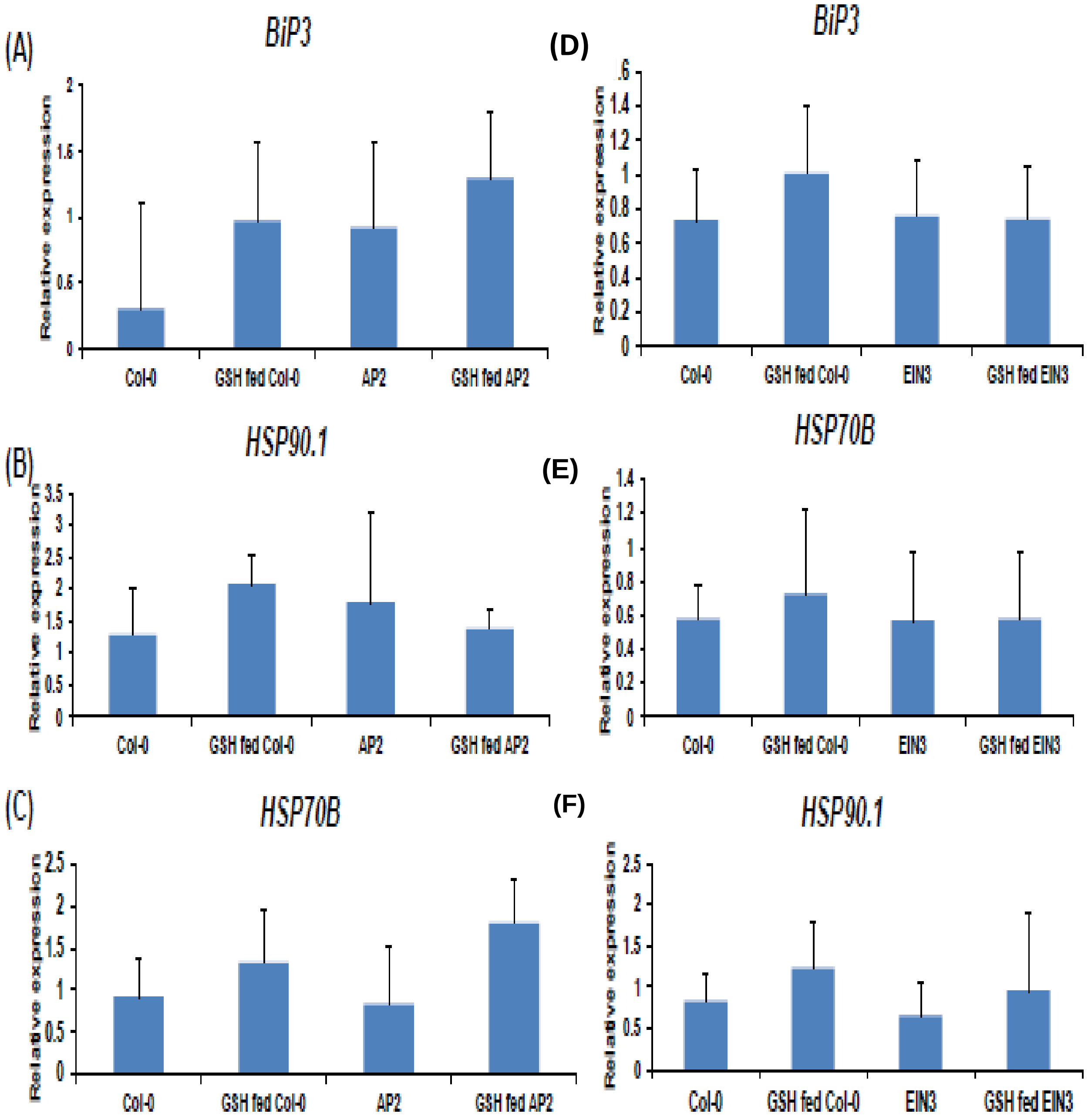

Supplementary Figure S8. **Effect on the expression of *HSPs* in GSH fed *Atap2* and *Atein3* mutant line.** (A), (B) and (C) revealed the expression of *BIP3*, *HSP70B* and *HSP90.1* in Col-0, GSH fed Col-0, *Atap2* and GSH fed *Atap2*. (D), (E) and (F) showed the expression of same HSPs in Col-0, GSH fed Col-0, *Atein3* and GSH fed *Atein3*. Data are presented as mean± SE (*n*=3). Results revealed no significant difference in the expression of *HSPs* between GSH fed Col-0 and mutants which indicated towards less role of APETALA2 and EIN3 TFs in inducing these HSPs in response to GSH feeding.

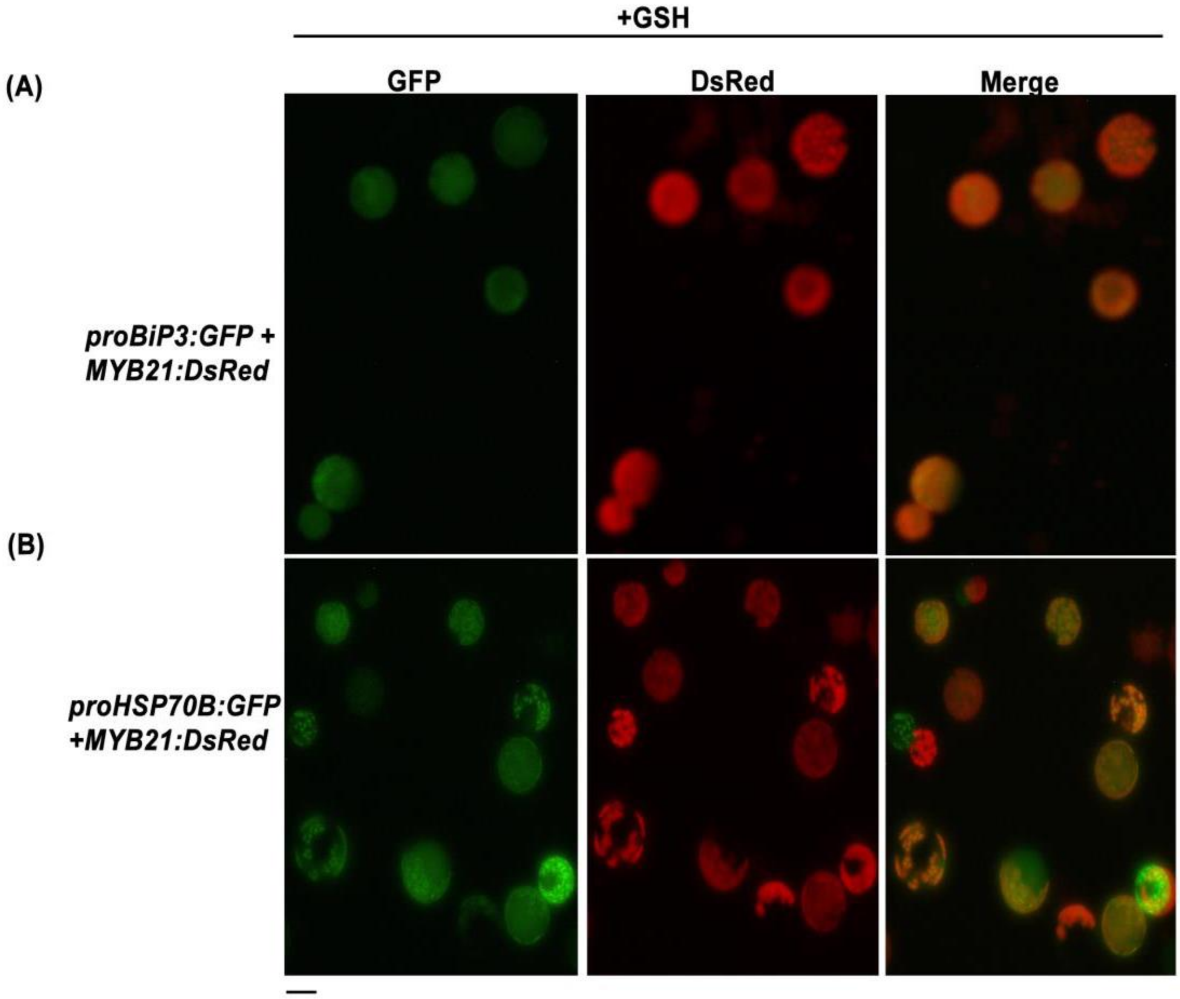

Supplementary Figure S9. Co-transfection of protoplasts Atmyb21 in GSH fed condition . (A) and (B) showed the elevated level of activation of promoters of *BiP3* and *HSP70B* after co-transfection of the protoplasts of *Atmyb21* with *proCAM35S:MYB21-DsRed*, *proBip3:GFP* and *proHSP70B:GFP* under GSH-fed condition. Promoter activation and *myb21* expressions are denoted by GFP and DsRed fluorescence. Black Bar: 20 µm

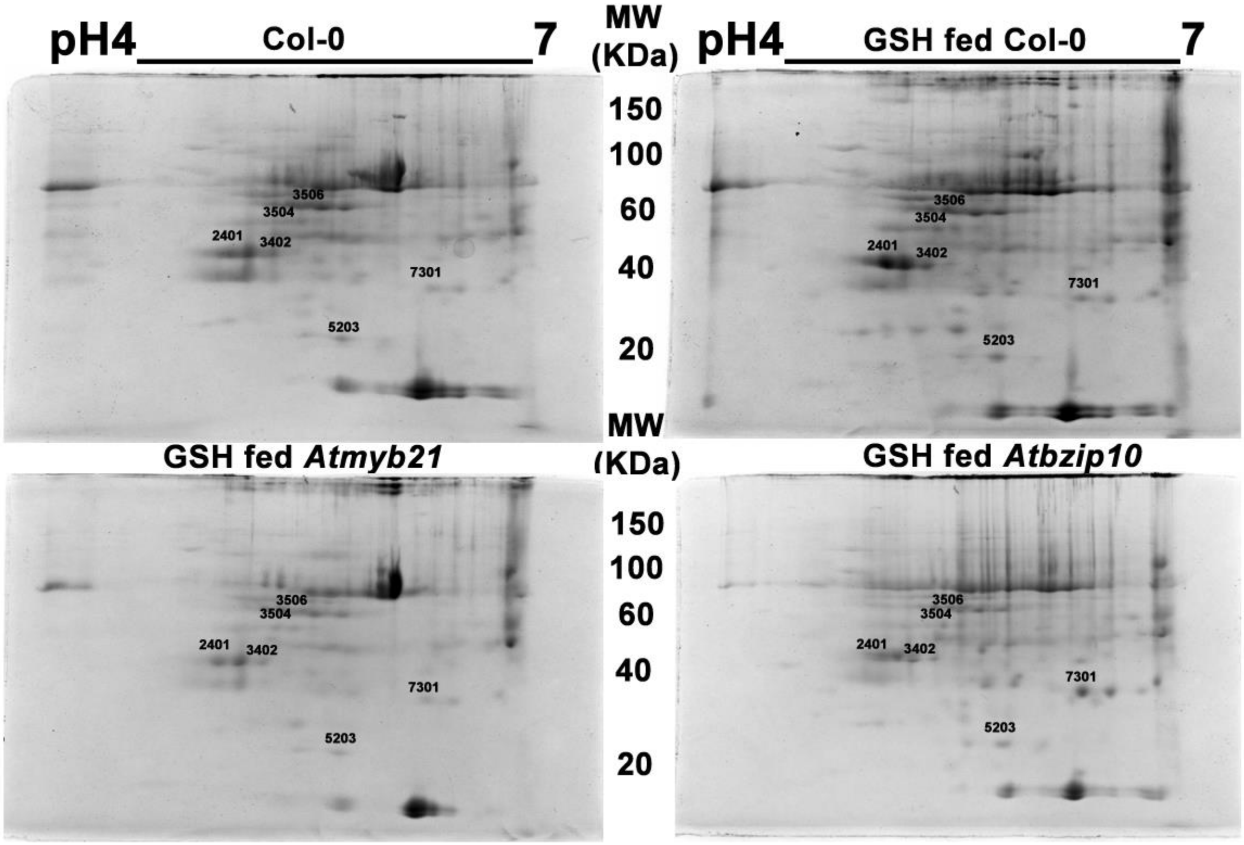

Supplementary Figure S10. 2DE gel picture of comparative proteomic analysis of Col-0, GSH fed Col-0 GSH fed *Atmyb21* and GSH fed *Atbzip10* mutants. Differentially expressed protein spots in these plant samples are denoted by protein SSP number

Table S1.: List of primers used in the quantitative and semiquatitative RT-PCR.

Genes	Forward Primer (5'- 3')	Reverse Primer (5'-3')
HSP90.1	ATGGCGGATGTTCAGATGGCT	TTCCCAAGTTGTTCACCAAAT
Actin	GGCTGATGGTGAAGATATTCAAC	CATTGTAGAAAGTATGATGCCAGA
HSP70B	ATGGCGACGAAATCAGAGAAA	CGGGGCCGGAAACAACCTTGA
MYB21	AGTTCTAGAATGGAGAAAAGAGGAGGAGGAA	ACTGGATCCTCAATTACCATTCAATAAATGCA-
BZIP10	GAATTCATGAACAGTATCTTCTCCATTG	CCATGGTTACCATGCTGAATCGTTCTGA

Table S2: Value of GFP fluorescence in (A) control protoplast(B) *proBiP3:GFP* transfected protoplasts (C) *proHSP70B:GFP* transfected protoplasts (D) *proHSP90.1:GFP* transfected protoplasts of Col-0, *pad2.1* and *AtECS1*.

(A) Control

No.	Area	Mean	Min	Max
1	3024	4	4	4
2	4087	4.016	4	5
3	4095	4.061	4	5
4	4422	4.038	4	5
5	5400	4.009	4	5
6	5751	4	4	4
7	4200	4.004	4	5
8	5293	4.036	4	5
9	5460	4.258	4	6
10	5350	4.19	4	5

(B) *ProBiP3:GFP* transfected protoplasts

Col-0

No.	Area	Mean	Min	Max	Mean/area	Normalized value
1	4352	17.723	4	71	0.004	444
2	6723	17.347	3	70	0.002	222
3	6278	15.7	4	58	0.002	222
4	5236	16.542	4	52	0.003	333
5	6825	13.631	4	52	0.001	111
6	5396	12.083	4	47	0.002	222
7	5400	13.46	4	48	0.002	222
8	4154	13.362	4	51	0.003	333
9	3660	14.449	4	51	0.0039	433
10	2800	10.71	4	37	0.0038	433
11	3224	14.05	4	47	0.004	444
					**Sum**	3419
					**Mean**	310

pad2.1

No.	Area	Mean	Min	Max	mean/area	normalized with control
1	6972	8.533	3	25	0.0012	133
2	8004	8.903	4	25	0.0011	122
3	5920	7.209	3	23	0.0012	122
4	5467	8.187	4	22	0.0015	166
5	5904	5.752	4	14	0.0009	100
6	6048	6.445	4	17	0.001	122
7	3363	8.339	3	19	0.0024	266
8	3355	5.727	4	13	0.0017	166
9	4960	7.657	3	18	0.00154	171
10	2805	8.882	3	19	0.0031	344
11	5568	5.525	4	12	0.00099	100
					**Sum**	1812
					**Mean**	164

AtECS1

No.	Area	Mean	Min	Max	Mean/area	Normalized with control
1	2867	24.593	4	84	0.0085	944
2	5236	28.806	5	105	0.0055	611
3	4480	27.373	3	97	0.006	666
4	4160	25.216	6	76	0.006	666
5	3283	36.744	5	102	0.011	1222
6	5184	26.989	6	98	0.005	555
7	5916	27.273	5	103	0.0046	511
8	5772	25.349	6	98	0.004	444
9	3538	28.662	3	87	0.008	888
10	3300	28.915	6	76	0.008	888
11	3416	29.49	5	87	0.008	888
					**Sum**	8283
					**Mean**	753

(C) *proHSP70B:GFP* transfected protoplasts

Col-0

No.	Area	Mean	Min	Max	Mean/Area	Normalized with control
1	5040	13.522	4	54	0.0026	288
2	6048	12.48	3	45	0.002	222
3	5986	12.293	4	55	0.002	222
4	6808	14.693	4	59	0.0021	227
5	7917	9.509	3	45	0.001	111
6	5256	17.466	3	61	0.0033	366
7	6120	15.943	4	59	0.002	222
8	8099	13.307	4	57	0.00164	182
9	5148	15.377	5	62	0.0029	322
10	4087	12.079	4	45	0.0029	322
					**Sum**	2484
					**Mean**	248.4

pad2.1

No.	Area	Mean	Min	Max	mean/area	Norrmalized with control
1	3776	8.461	4	19	0.0022	244
2	5712	8.37	4	19	0.0014	155
3	3250	8.842	4	22	0.0027	288
4	3770	6.899	3	17	0.0018	200
5	3744	7.889	4	29	0.0021	233
6	5041	8.143	4	26	0.0016	177
7	4620	8.61	3	35	0.0018	200
8	3306	8.42	4	28	0.0025	277
9	4392	7.068	4	22	0.0016	177
10	4662	7.152	4	27	0.00153	170
					**Sum**	2121
					**Mean**	212.1

AtECS1

No.	Area	Mean	Min	Max	mean/area	Normalized with control
1	5040	20.966	5	91	0.0041	455
2	5256	24.604	5	109	0.0046	511
3	4221	29.484	4	99	0.0069	766
4	8742	24.426	4	93	0.0027	300
5	5256	19.954	4	75	0.0037	411
6	6084	21.504	4	90	0.0035	388
7	6804	18.932	4	73	0.0027	300
8	3780	21.097	5	80	0.00558	620
9	3216	22.928	5	87	0.0071	788
10	5328	15.6	5	104	0.0029	322
					**Sum**	4861
					**Mean**	486

(D) *proHSP90.1:GFP* transfected protoplasts

Col-0

No.	Area	Mean	Min	Max	Mean/Area	Normalized with control
1	4160	12.288	3	51	0.0029	322
2	5320	12.2	4	48	0.0023	255
3	5688	10.913	4	27	0.0019	211
4	5621	9.815	4	32	0.00174	193
5	5550	12.434	3	44	0.00224	248
6	6192	13.882	4	36	0.00224	248
7	6804	13.187	4	53	0.00193	214
8	7565	11.938	4	46	0.00157	174
9	6552	10.802	3	37	0.00164	182
10	5700	9.745	4	32	0.0017	188
11	5476	10.359	4	35	0.00189	210
12	4588	12.192	4	40	0.00265	294
					**Sum**	2739
					**Mean**	228

pad2.1

No.	Area	Mean	Min	Max	Mean/Area	Normalized with control
1	4221	9.136	4	34	0.00216	240
2	6090	9.467	3	24	0.00155	172
3	8008	9.073	3	35	0.0011	122
4	5390	10.054	4	23	0.00195	216
5	9936	7.863	4	25	0.00079	87.77
6	5040	10.017	4	31	0.0019	211
7	6450	7.872	3	30	0.00122	135
8	5780	6.609	4	25	0.00114	126
9	5893	6.923	4	34	0.00117	130
10	2592	9.13	3	28	0.0035	388
					**Sum**	1827.77
					**Mean**	182.777

AtECS1

No.	Area	Mean	Min	Max	Mean/Area	Normalized ratio
1	8526	24.799	3	93	0.0029	322
2	6150	29.04	5	103	0.0047	522
3	6560	27.226	5	95	0.0041	455
4	4209	26.594	6	77	0.0063	700
5	4636	27.286	5	105	0.0059	655
6	4615	25.776	4	97	0.0055	611
7	3960	26.022	5	69	0.006571	722
8	4828	21.633	5	66	0.00448	533
9	3540	29.735	6	77	0.0083	922
10	3480	21.069	5	81	0.006	666
					**Sum**	6108
					**Mean**	610.8
